# Global profiling of phytohormone dynamics during combined drought and pathogen stress in *Arabidopsis thaliana* reveals ABA and JA as major regulators

**DOI:** 10.1038/s41598-017-03907-2

**Published:** 2017-06-21

**Authors:** Aarti Gupta, Hiroshi Hisano, Yuko Hojo, Takakazu Matsuura, Yoko Ikeda, Izumi C. Mori, Muthappa Senthil-Kumar

**Affiliations:** 1 National Institute of Plant Genome Research, Aruna Asaf Ali Marg, JNU campus, New Delhi, 110067 India; 2 Institute of Plant Science and Resources, Okayama University, Kurashiki, 710-0046 Japan

## Abstract

Global transcriptome studies demonstrated the existence of unique plant responses under combined stress which are otherwise not seen during individual stresses. In order to combat combined stress plants use signaling pathways and ‘cross talk’ mediated by hormones involved in stress and growth related processes. However, interactions among hormones’ pathways in combined stressed plants are not yet known. Here we studied dynamics of different hormones under individual and combined drought and pathogen infection in *Arabidopsis thaliana* by liquid chromatography-mass spectrometry (LC-MS) based profiling. Our results revealed abscisic acid (ABA) and salicylic acid (SA) as key regulators under individual drought and pathogen stress respectively. Under combined drought and host pathogen stress (DH) we observed non-induced levels of ABA with an upsurge in SA and jasmonic acid (JA) concentrations, underscoring their role in basal tolerance against host pathogen. Under a non-host pathogen interaction with drought (DNH) stressed plants, ABA, SA and JA profiles were similar to those under DH or non-host pathogen alone. We propose that plants use SA/JA dependent signaling during DH stress which antagonize ABA biosynthesis and signaling pathways during early stage of stress. The study provides insights into hormone modulation at different time points during combined stress.

## Introduction

Under field conditions, drought often in combination with pathogen inflicts greater crop yield loss^1, 2^. The net impact of combined stresses on plant health and yield is different from those imposed by individual stresses^3^. The net impact of combined stress often depends on synergistic or antagonistic interaction between the two stressors^1^. Such interactions are dictated by type of pathogen^4, 5^, time and order of occurrence^6^ and intensity of two stressors^4, 7, 8^, age^9^ and genotype of the plant^10^. Recent studies on combined stressed plants revealed that the response and state of plants under combined stress is different from that under individual stresses^3, 6, 7, 11–13^. However, these plants also exhibit a few common molecular responses which are reminiscent of either of the individual stresses^3, 6, 7, 11–13^. The reminiscent responses equip plants for upcoming new or additional stresses through signaling and crosstalk. Considering the existence of unique and reminiscent responses under combined stress, it is proposed that plants experience a new state of stress when exposed to combined stress. Such interactions that regulate plant responses to combined stress and brings in tolerance could thus be understood only by exploring combined stressed plants.

One of the important mediators of interaction between biotic and abiotic stresses is phytohormones and derived signaling. The interplay between hormone levels and signaling networks make them ideal candidates for mediating combined stress responses. So far, plant adaptive strategies to individual pathogen and drought stresses have been extensively studied. Largely, abscisic acid (ABA), salicylic acid (SA) and jasmonic acid (JA), are key mediators of drought and pathogen stress responses. Growth process regulating hormones cytokinin (CK), auxin, ethylene (ET), gibberellins (GA) and brassinosteroids (BR) are also reported to play secondary role in mediating the stress responses^14–20^. SA is known to play major role in plant defense against biotrophic pathogens and JA and ET are generally associated with defense against necrotrophic pathogens^21^. Drought stress responses are known to be mediated by ABA^22^. Though individual stress-based data has been gathered over the years for ABA, SA, JA and growth regulating hormones, their role and regulation in combined stress is still not unraveled. The interaction between drought and pathogen phytohormonal signaling pathways can be positive and negative and can thus accordingly influence the net impact caused by combined stress. Positive interaction between two signaling pathways may lead to increased tolerance under the combined stress. The negative interactions on the other hand lead to dominance of one stressor over other^23^. For example, ABA treatment or simulated drought stress antagonizes SA -mediated defenses and imparts susceptibility of plants towards pathogens^24, 25^. However, studies also showed positive interaction between ABA and SA signaling pathways that lead to enhanced stomatal closure and resulted in drought stress tolerance^26–29^. Similarly, the SA- JA- crosstalk has been dose dependent where synergism between these two is found to be at lower endogenous concentrations which reverses to antagonism at higher concentrations of either of the two^30^. In another instance, *Arabidopsis thaliana* quadruple mutants deficient in DELLA protein (repressors of GA-mediated responses) exhibited reduced expression of the defense gene *PDF1.2* which is a marker gene of JA-mediated signaling. Such an antagonism between GA and JA resulted in increased susceptibility of the plants to the necrotrophic fungus *A. brassicicola* ^31^. Additionally, drought stress lead to reduced indole-3-acetic acid (IAA, auxin) content^32^, however, increased auxin levels were noted upon pathogen infection^16^. Looking at other examples cited in Supplementary Table S1, it is possible that positive and negative crosstalk between different stress hormones regulates interaction between drought and pathogen stress signaling^18, 33–37^.

Crosstalk among different hormones further lead to complex signaling pathways when host pathogen exploits and modulates phytohormone levels to combat plant defense and establish itself in plant cell^19, 38^. For example, *P. syringae* produces a virulence factor coronatine, a JA mimic, which suppresses SA-mediated defenses in plants to facilitate infection^39^. Additionally, HopAM1 effectors also modulate pathogenicity of avirulent pathogen on drought stressed plants by mediating ABA signaling^40^. In an another instance ABA pretreatment lead to increased growth of the AvrXccC8004-deficient strain^41^. These arguments suggest that under natural field conditions the infection of a non-host pathogen in plants can be different during drought stress. Not only this, the phytohormone levels tend to be different when the plant abiotic and biotic stress pathways collide during combined stress.

The cited arguments thus reflect that the phytohormone modulations under combined drought and foliar bacterial pathogen stress could not be derived and justified from single stress studies. This presents the need to study the phytohormone signaling under combined drought and pathogen stress in order to understand plant responses to the combined stress conditions. In the present study, we exposed *Arabidopsis thaliana* to combined drought stress and host or non-host foliar pathogen. We studied phytohormone modulations at different time of occurrence of two stressors (drought and host) and during pathogen infection on drought recovering plant. ABA, SA, JA, CK, GA and auxin were profiled in *A. thaliana* leaves using liquid chromatography-mass spectrometry (LC-MS) at different time points of 2, 8 and 24 h post combined stress treatment. We comprehensively present different hormone levels at 8 h post stress treatment. In addition, based on microarray data, we also correlate the hormone content at early time point at 8 h and late time point at 24 h post treatments in regulating the derived signaling or hormone biosynthesis respectively.

## Results

### Combined stress imposition and features of phytohormone profile

*Arabidopsis thaliana* was exposed independently to drought (D), host (H) and non-host (NH) pathogen and to their combinations namely, drought with host pathogen (DH) and drought with non-host pathogen (DNH) to study the hormonal profile under each stress (Supplementary Fig. S1). Since time of occurrence of pathogen stressor in the combined drought and pathogen (DH) stress may differentially influence the plant response, the host pathogen inoculation prior to drought stress (HD) treatment was also studied. Additionally, the plants elicit robust defenses under non-host treatment (over host infection) without disease. In this purview, combined DNH treatment provides a conducive model to study plant defense response under combined stress. Moreover, the DNH treatment allows understanding of host effectors mediated phytohormone modulation during combined stress. Plant responses instigated by drought recovery are different from drought stress and non-stressed state and resultantly can aid or antagonize plant interaction with pathogen. In this view, we also studied phytohormone modulation in plants under pathogen infection after drought recovery (DRH).

The impact of water withholding on plants was ascertained by estimating the relative water content (RWC) of leaf. Both soil and leaf water status measurements indicated gradual decline of soil moisture content and leaf RWC in treatments involving drought stress (D, DH, DNH) (Supplementary Fig. S1). The DRH treatment on the other hand exhibited gradual saturation of soil moisture content and increase in RWC (Supplementary Fig. S1). After 24 h of host pathogen inoculation, *in planta* bacterial multiplication was noted (over initial inoculum at 0 h post treatment, hpt) in H, DH and DRH plants indicating host pathogen multiplication (Supplementary Fig. S1). Time of pathogen inoculation during combined stress has been shown to drive the outcome of stress interaction as reflected *in planta* bacterial multiplication. Our results on *in planta* bacterial numbers indicated that combined stressed plants (DH and DRH) led to reduction of *in planta* bacterial numbers as compared to single stressed H only plants^8^. However, in HD plants the *in planta* bacterial numbers did not change over plants under individual host pathogen (for long duration; 5 days after inoculation, H5) plants (Supplementary Fig. S1).

**Figure 1:**
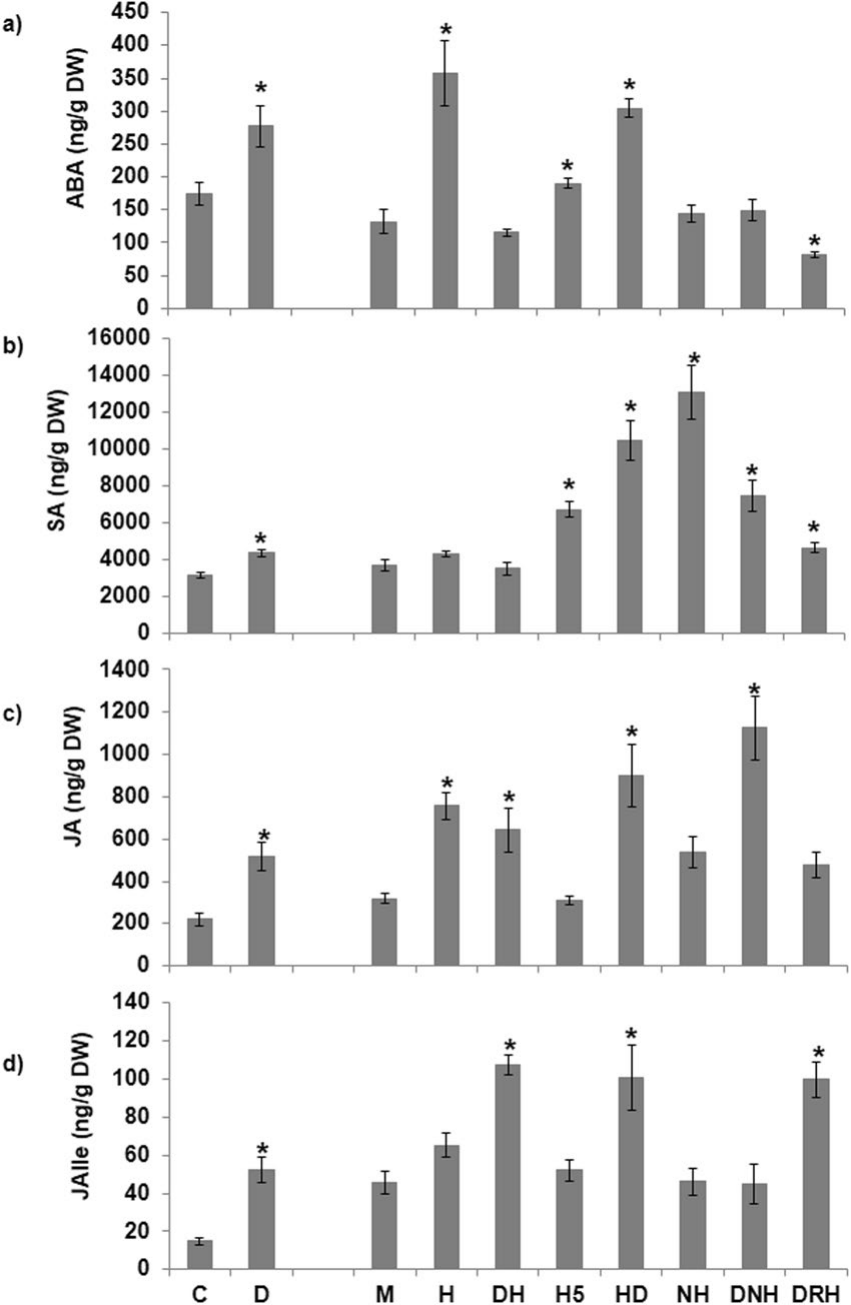
Modulation in phytohormones levels in leaves of *Arabidopsis thaliana* exposed to individual and combined stresses. Leaf samples were harvested at 8 hours post pathogen inoculation/combined stress treatment (hpt), lyophilized and were used for LC-MS based phytohormone quantification. Plants were subjected to drought (D), host pathogen (H, *Pseudomonas syringae* pv. tomato DC3000) or H5 (host pathogen treatment for 4.8 days), non-host pathogen (NH),combined drought followed by pathogen (DH) or non-host pathogen (DNH), combined pathogen followed by drought stress (HD), combined drought or pathogen infection during drought recovery (DRH) along with their respective controls (D over absolute or H, H5, NH, DH, HD, DNH and DRH over mock control). All leaves were used to quantify hormone concentrations. Absolute levels of ABA (**a**), SA (**b**), JA (**c**) and JA-Ile (**d**) are presented in terms of ng/g dry weight of the samples. Each data point represents the mean of at least three independent replicates and error bars denote standard error of mean. Significant difference in hormone levels in individual drought stressed samples was assessed over absolute control. Significant difference in hormone levels in individual H, H5, NH or combined HD, DNH and DRH treatments was assessed over mock control. Significant difference between different treatments and controls was analyzed by student’s *t*-test. Asterix represent significant difference between treatment and control at *p* < 0.05. Comparison among individual and combined stresses is presented in terms of fold change over their respective controls in Supplementary Figs S4–S6. Raw values for phytohormone concentrations at 2, 8 and 24 hpt, number of replicates and calculated standard error of mean over respective controls are presented in Supplementary File S1.

**Figure 2:**
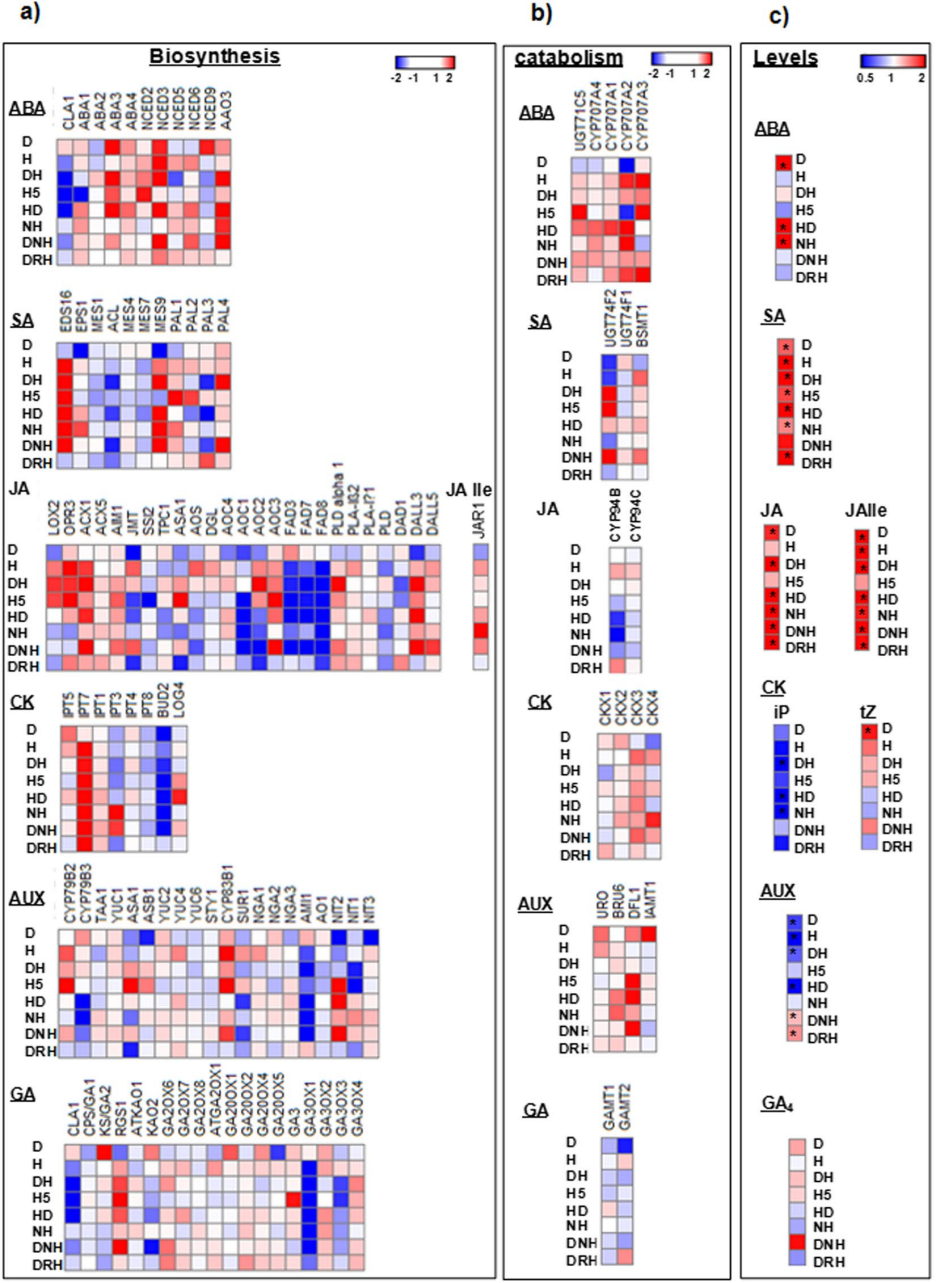
Transcript expression pattern of hormone biosynthesis related genes and hormone levels in *A. thaliana* under individual and combined stresses. Transcriptome analysis of hormone biosynthesis and catabolism related genes was correlated with endogenous phytohormone concentrations at 24 hpt under different individual and combined stress treatments. Heat maps reflect transcript expression pattern of phytohormone biosynthesis (**a**) and catabolism (**b**) associated genes. Previously, *A. thaliana* plants were exposed to different stress treatments (same as the one used in present study) and leaf samples were harvested at 24 hpt. Microarray experiment was conducted with these samples and microarray data was submitted to GEO NCBI (accession no. GSE79681). Differentially expressed genes were identified in stressed samples over control using Expression Console and Transcriptome Analysis Console (Affymetrix, California, USA). Genes involved in hormone biosynthesis and catabolism were identified through manual curation in literature and Arabidopsis Hormone Database 2.0 (http://ahd.cbi.pku.edu.cn/). Full details of genes names and corresponding ID are provided in Supplementary File S2. Heatmaps presented in (**c**) indicate fold change in hormone levels under different treatments over control at 24 hpt. Significant difference in treatments over control was calculated using Student’s *t*-test and *indicates significant change in hormone levels over control at p ≤ 0.05. Color bar ranging from red to blue represents up-regulation and down-regulation in transcript expression or phytohormone concentrations. Raw values of phytohormone profile with standard error of means and number of replicates are mentioned in Supplementary File S1. D; drought, H; host pathogen Pst DC3000, DH; combined drought and host pathogen stress, H5; host pathogen Pst DC3000 infection for 5 days, HD; combined host pathogen and drought stress, NH; non-host Psta, DNH; combined drought and non-host, DRH; combined drought recovery host pathogen stress.

Phytohormone profiling for abscisic acid (ABA), salicylic acid (SA), jasmonic acid (JA), and its amino acid conjugate JA-isoleucine (JA-Ile), cytokinin (trans-zeatin, tZ and isopentenyl adenine, iP), auxin (indole acetic acid, IAA) and gibberrelic acid (GA_4_) was studied in individual D, H, H5, NH, and combined DH, HD, DNH and DRH stressed plants at different time points post treatment using LC-MS based hormone quantification (Supplementary Figs S2 and S3). Dendrogram derived analysis (based on Pearson correlation coefficient) of phytohormone values under different treatments showed high correlation among treatments involving pathogen inoculation under well-watered conditions (H, H5 and DRH) and the drought stress treatment associated closely (Supplementary Fig. S2). Under drought stress, we noticed significant increase in ABA, SA, JA, JA-Ile, and tZ class of cytokinin levels over control (Fig. 1; Supplementary File S1). Similarly, the host pathogen inoculation (H) led to initial induction of ABA, JA and auxin (IAA) at 8 hpt (Fig. 1; Supplementary Fig. S9; Supplementary File S1). Individual drought and host pathogen stress data was found to be in corroboration with the hormone modulation reported in literature (Supplementary Fig. S7). These results reflected authenticity of the stress imposition protocol and relatedness of different treatments. We did not observe any significant alterations in GA_4_ level under any of the individual or combined stress treatments (Fig. 2; Supplementary Figs S8 and S9; Supplementary File S1).

### Plants under combined drought and host pathogen stress differentially modulates phytohormone levels over individual stress

Phytohormones modulation were assessed under individual drought and host pathogen and combined drought and host pathogen treatments and further expressed as fold change over control. We also analyzed transcriptome profile of hormone biosynthesis, catabolism, transport, receptor and signaling pathway related genes using microarray data available in the lab (GEO NCBI accession no. GSE79681). The early induction of hormone levels at 8 hpt could possibly modulate the hormone signaling cascade at later time point (24 hpt) (Supplementary Fig. S9). From this perspective, we associated the hormone biosynthesis and catabolism pathway genes’ expression with levels of hormones at 24 hpt (Fig. 2).

Under combined DH treatment the ABA levels were not induced over control at both time points i.e., at 8 and 24 hpt. Although, ABA levels in combined DH stressed plants were reduced compared to host pathogen at 8 h time point, later at 24 hpt, the H stressed plants exhibited drastic reduction in ABA levels and were at par with combined stressed and control plants (Figs 1a, 2; Supplementary Figs S4a and S9). Such a profile can be considered as tailored response under combined stressed plants. Combined stressed plants exhibited high SA levels at 2 and 24 hpt but no change at 8 hpt in comparison to control (Figs 1b, 2; Supplementary Figs S4b, S8 and S9; Supplementary File S1). With reference to the individual D and H stress treatments, the DH stressed plants exhibited reduced SA levels at 8 hpt. SA levels in combined stressed plants were induced to high levels at 24 hpt over individual D and H plants (Supplementary Fig. S4b). The levels of JA or its active form JA-Ile were induced under combined stress in comparison to control at both time points (Figs 1c,d and 2; Supplementary Fig. S4c and Supplementary File S1). In DH plants, JA levels remain unchanged at 8 hpt over D and H stress, however the levels were markedly induced over individual stresses at 24 hpt (Supplementary Fig. S4c). In comparison to the individual stresses, JA-Ile, the functionally active form of JA, levels remained unchanged at 8 hpt but at 24 hpt, it was induced to high levels in DH plants over H stressed plants (Supplementary Fig. S4c). Notably, the JA/JA-Ile levels were highest during D stress followed by combined DH stress and H stress (Fig. 1c,d). Overall, combined DH stressed plants exhibited unique trend in terms of ABA levels. On the other hand similar trend in SA or JA levels across individual and combined stressed plants was regarded as shared response (Supplementary Fig. S4).

Further, combined DH stressed plants displayed unchanged or reduced isopentenyl adenine (iP) levels at 8 and 24 hpt respectively as opposed to the significant induction of trans-zeatin (tZ) at 24 hpt over control (Fig. 2; Supplementary Fig. S9; Supplementary File S1). Auxin levels were not altered at 8 hpt, but were elevated at 24 hpt in comparison to the control (Fig. 2; Supplementary Fig. S9; Supplementary File S1). The endogenous GA levels were undetectable in combined DH stressed plants (Fig. 2; Supplementary Fig. S9 and Supplementary File S1).

**Figure 3:**
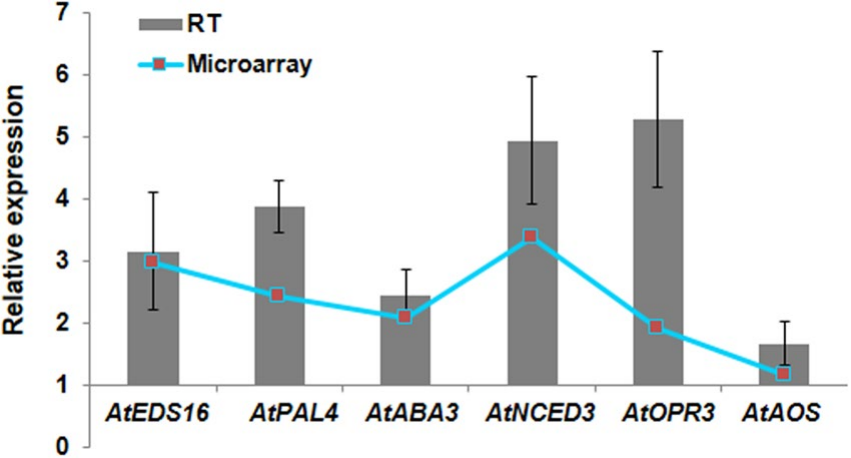
Validation of transcript expression pattern by RT-qPCR. Transcript expression profile of key genes involved in hormone biosynthesis obtained from microarray experiment was validated by RT-qPCR. Graph represents relative expression of the genes under combined DH stress. Bar and line graphs represent expression profile obtained from RT-qPCR and microarray experiments respectively. In RT-qPCR experiment, *AtActin2* was used as reference gene for data normalization. Fold change was calculated over mock control. Each bar represents average of four biological and two technical replicates ± SEM.

During combined DH stress we noted in microarray data that expression of ABA biosynthesis genes *CAI* and 9-cis-epoxycarotenoid dioxygenase (*NCED5*) were down-regulated and *NCED3* and abscisic aldehyde oxidase 3 (*AAO3*) genes were up-regulated. However, the catabolism pathway related genes expression remain unchanged (Fig. 2). The induction of few biosynthesis genes in DH plants possibly contribute to the non-induced basal levels of ABA in combined stressed plants. Furthermore, the genes involved in ABA transport and negative regulators of ABA signaling were up-regulated (Supplementary Fig. S9). The increased expression of genes encoding for PP2CAs (negative regulators of ABA signaling) and SnRK2s (positive regulators of ABA signaling) hints toward activated ABA dependent signaling in DH stressed plants. The SA biosynthesis pathway genes involving enhanced disease susceptibility (EDS, *EDS16*) and phenylalanine ammonia-lyase (PAL, *PAL4*) branch^42–44^ were up-regulated and this is consistent with increased SA levels at 24 hpt (Figs 2, 3 and Supplementary Fig. S10). Transcript profile of *AtPAL1* and *2* revealed their unchanged expression under DH stress over control and suggest the specific involvement of *AtPAL4* in modulation of SA levels in these plants (Supplementary Fig. S10). It is perceived that the unchanged levels of SA (over control) at 8 hpt possibly caused activation of genes involved in SA transport and perception. The downstream PR mediated signaling and *WRKY53* and *WRKY70* genes expression were up-regulated (involved in defense signaling, Supplementary Fig. S9). Similarly, JA biosynthesis genes, allene oxide synthase (*AOS*) and 12-oxophytodienoate reductase 3 (*OPR3*) were up-regulated along with induced JA/JA-Ile levels at 24 hpt (Figs 2 and 3). From the present results, it is apparent that the JA level induced at 8 hpt did not instigate any change in expression of *GTR1* (mediates JA transport) and *COI1* (dependent perception) (Supplementary Fig. S9). The JA levels at 8 hpt however, lead to activation of JAZs and *WRKY70* mediated signaling pathway (Supplementary Fig. S9). The results obtained from microarray experiment were validated by q-PCR for key genes involved in hormone biosynthesis at 24 hpt. Accordingly, q-PCR based up-regulated expression profile of the rate limiting hormone biosynthetic genes; *NCED3*, *ABA3*, (ABA^45^), *AOS* and *OPR3* (JA^46^) *EDS16* and *PAL4* (SA^47^) under combined DH stress was in line with microarray results (Fig. 3, Supplementary File S4).

### Temporal regulation of drought stress mediated hormone changes in combined stressed plants

Previously, we demonstrated the influence of order of occurrence of two stressors on stress interaction and plant physiology during combined stress^6, 8^. In order to further dissect the hormonal crosstalk and regulation underlying such phenomenon of temporal influence of stressor, we studied the comparative phtytohormone profile under DH and HD stress treatments. For HD stress imposition, pathogen inoculation preceded the commencement of drought stress along with the respective long duration pathogen stressed plants (H5) maintained as its individual stress control. In this regard, we quantified various phytohormone levels in HD plants. The ABA, SA, JA/JA-Ile in HD combined stresses plants were induced over control plants at both early and late time points (Figs 1, 2; Supplementary Fig. S9 and Supplementary File S1). In comparison to the individual stress, the ABA accumulated more at 8 hpt during HD stress (Supplementary Fig. S4d). However, at 24 hpt, ABA levels remained unchanged over D, but increased over individual H5 stress (Supplementary Figs S4d and S9). SA levels in HD plants were also different from individual stress at early time point but exhibited no change at 24 hpt over individual stresses (Supplementary Figs S4e and S9). JA levels were increased over H5 only stress at both times but JA-Ile was increased only at 24 hpt time point (Supplementary Figs S4f and S9). Thus inoculation of pathogen prior to drought stress in combined HD stressed plants has led to accumulation of ABA and SA at both early and late time points as opposed to the no change in ABA levels and late induction of SA levels (at 24 hpt) in combined DH plants.

Additionally, the genes involved in ABA biosynthesis and catabolism were up-regulated inciting the ABA levels in HD stressed plants at 24 hpt (Fig. 2; Supplementary Fig. S9). The accumulation of ABA at 8 hpt could have activated the expression of *ABCG40* gene (an ABA transporter involved in cellular uptake of ABA). The expression levels of genes encoding ABA receptors GUN5, PYR1 and RCAR3 was down-regulated while the genes ABF2, ABI2/5 and AFP1/2/3 mediating ABA signaling were up-regulated (Supplementary Fig. S9). High SA levels at 24 hpt were collinear with activation of EDS16 dependent SA biosynthesis without change in SA catabolism genes (Fig. 2). HD stressed plants exhibited up-regulation of SA transporter and signaling genes (Fig. 2). High levels of JA/JA-Ile at 24 hpt were consistent with down-regulation of *CYP94B* (a JA catabolism gene, Fig. 2). JA/JA-Ile accumulation at 8 hpt was opposed to the observed down-regulation of gene encoding for GTR1, a JA/JA-Ile transporter and COI1, a JA receptor but must have instigated the activation of *JAZ1*, *JAZ11* and *WRKY70* genes mediating JA signaling pathway (Supplementary Fig. S9).

DH and HD hormone and transcriptome profile indicated that high ABA level in the latter case could be the factor mediating differential response of combined stressed plants towards the *in planta* bacterial multiplication. This is also supported by the observed collinearity in high ABA levels and *in planta* pathogen multiplication under H only stressed plants.

### Phytohormone modulation under combined drought and non-host pathogen treatment

During non-host pathogen infection, plant defend itself while eliciting vigorous defense responses. In this purview, combined DNH treatment provides a favorable experimental platform to study plant defense response under combined stress. Besides, understanding plant responses towards combined drought and pathogen stress is obstructed by the pathogen inflicted alteration of phytohormones which not only influence the resultant phytohormone profile but also the downstream signaling^14^. The contribution of pathogen molecule towards stress interaction during combined stress can be better studied by combining non-host pathogen with drought. Here we compared phytohormone profile of combined DNH stressed plants with individual D and NH stresses and also associated with the transcriptome data. We observed no change in ABA levels under DNH stress over control at either of the time points (Figs 1a, 2; Supplementary Fig. S5, S9 and Supplementary File S1). In comparison to the individual NH treatment, DNH treated plants exhibited similar ABA levels at 8 hpt but remarkably different from highly induced levels of ABA under NH treatment (Supplementary Fig. S5a). SA level was induced over control but it was similar to individual stresses except reduction observed at 8 hpt over NH (Figs 1b, 2; Supplementary Figs S5b, S9 and Supplementary File S1). JA levels were also enhanced over control plants. While in comparison to the D stress, JA profiles at 8 hpt are induced to high levels but at 24 hpt was similar to the individual stresses (Figs 1c, 2; Supplementary Figs S5c, S9 and Supplementary File S1). The JA-Ile concentration was not induced at 8 hpt (over control) and was similar to the NH treatment (Figs 1d, 2; Supplementary Fig. S5c, S9 and Supplementary File S1). At 24 hpt, on the other hand, the differential accumulation of JA-Ile was seen under individual and combined stress (Supplementary Fig. S9 and Supplementary File S1).

We observed up-regulation of *NCED3* and *AAO3* which likely lead to the observed basal levels of ABA in DNH plants (Fig. 2). Also the SA biosynthesis genes *EDS16*, *MES9* and *PAL4* and SA catabolizing enzyme encoding gene *UGT74F2* were up-regulated in DNH plants (Fig. 2). This is in line with the observations made on DH stressed plants. Genes involved in JA biosynthesis *ACX1*, *AOC3* and *DALL3*/5 were also up-regulated and were similar to the observations made on DH plants (Fig. 2). In DNH plants, genes encoding ABA transporter ABCG40 was down-regulated while for SA transporter EDS5 was up-regulated (Supplementary Fig. S9). DNH plants showed down-regulation of genes encoding ABA receptors GUN5, PYR1 and RCAR3 similar to the DH plants. DNH plants exhibit up-regulation of SA receptors, NPR3 and NPR4 (Supplementary Fig. S9). The observed pattern for genes involved in ABA- mediated signaling was also similar in DNH and DH plants (Supplementary Fig. S9). Although the SA mediated signaling genes *PRB1*, *PR1*, *WRKY18* or WRKY54 or *WRKY58* was specifically up-regulated in DNH plants over DH plants, the expression of WRKY53/70 was found to be similar (Supplementary Fig. S9). This indicates more robust SA mediated defense in DNH plants which needs to be probed further. Based on transcriptome data we noted that DNH plants exhibit different JA response over DH plants.

Overall, we could see similar un-induced levels of ABA at early hours of combined stress treatments and similar transcriptome in DH and DNH plants. Taken together, ABA seemingly regulates the stress interaction during DH, HD and DNH combined stress treatments.

### Hormone modulation in plants inoculated with pathogen while recovering from drought stress

Previously it has been established that the plant responses to pathogen under non-stress, drought and drought recovery are different. So it was worthwhile to decode underlying phytohormone alterations in DRH plants. DRH treatment lead to significant reduction in ABA levels at 8 hpt but did not cause any change at 24 hpt over control and individual H stress (Figs 1a, 2; Supplementary Figs S6a, S9 and Supplementary File S1). No change in SA levels was observed at 8 hpt but significantly increased SA levels were observed in DRH stressed plants over control and H stress at 24 hpt (Figs 1b, 2; Supplementary Figs S6b, S9 and Supplementary File S1). Additionally, no change in JA levels was noted at 8 hpt but it increased at 24 hpt in DRH plants over control (Figs 1c, 2; Supplementary Figs S6c, S9 and Supplementary File S1). JA-Ile levels increased at 8 and 24 hpt over control and at 8 hpt over H stress (Figs 1d, 2; Supplementary Figs S6c, S9 and Supplementary File S1). Corresponding to reduced ABA levels, ABA catabolism genes *CYP70A2* and *CYP70A3* were highly up-regulated (Fig. 2). The ABA transporter and receptors were down-regulated but signaling genes were up-regulated (Supplementary Fig. S9). Expression profile of ABA transporters, receptors and signaling genes studied here was similar to H treatment. As opposed to its up-regulated expression in H plants, *ABCG40* gene expression was down-regulated in DRH stressed plants (Supplementary Fig. S9). *PAL3* gene involved in SA biosynthesis was up-regulated while *EDS16* mediated SA biosynthesis was activated in H stress (Fig. 2). The characteristic SA signaling genes namely, *EDS1*, *PAD4*, *NPR1* and *NPR2* remained unaltered under DRH treatment against up-regulated expression under H stress (Supplementary Fig. S9). However, the downstream signaling pathway genes viz., various PRs (*PR1*) and WRKYs (*WRKY53*) were similarly up-regulated in both DRH and H stresses (Supplementary Fig. S9). JA biosynthesis genes encoding JMT, ASA1, AOC2 and FAD8 were down-regulated while expression of catabolism genes encoding CYP94B/C remained unchanged (Fig. 2). Though the expression of genes encoding GTR1 transporter and COI1 receptor remain unchanged, the expression of *JAZ1* and *JAZ10* was oppositely regulated (up-regulation of *JAZ1* and down-regulation of *JAZ10*) over control (Supplementary Fig. S9).

Altogether, the presented results explain that the response of plant to each combined stress is different and thus it depends on the nature of the pathogen, and time of occurrence of each stressor (Supplementary Fig. S11).

## Discussion

In this manuscript, we have studied four different scenarios of stress interactions namely drought followed by host pathogen and pathogen infection on drought stressed plants, allowing the influences of each factor to be separated, drought and non-host, and pathogen inoculation on drought recovering plants. Based on hormone and transcript profile we identified the changes in hormonal pathway under these stress interactions and compared them with similar information from individual stress experiments. In the past, pathways which reveal role of hormone crosstalk and their mediated signaling were proposed based on information derived from individual stress studies^48^.

**Figure 4:**
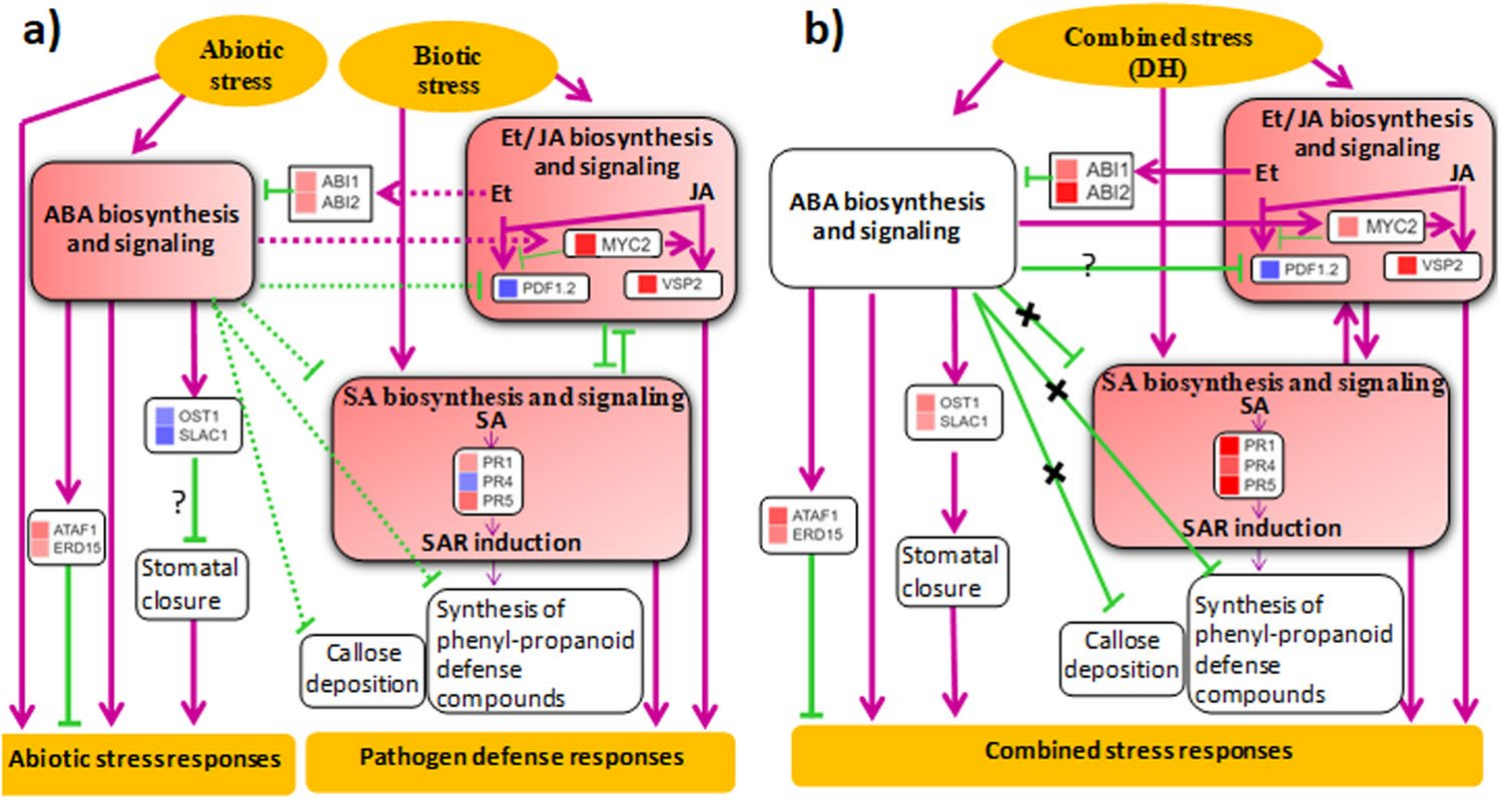
Schematic diagram illustrating the hormonal network under individual drought and host pathogen and their combination. The hormone and transcriptome profile data was integrated to redraw the model from Atkinson and Urwin (2012). The diagram depicts crosstalk between different hormones during individual and combined stress. The role of plant hormones in regulating the interaction between biotic and abiotic stress is presented based on individual stress data (**a**). The role of plant hormones in regulating combined drought and host pathogen stress (DH) is presented based on combined stress data (**b**). Red color boxes indicate elevation in hormone content and white boxes indicate no change over control. Green arrows show induction or positive regulation, while yellow bars show inhibition or repression of gene or process. The crosstalk between abiotic and biotic stress response is shown as dashed arrows or bars. Cross signs represent inhibition of the step in absence of hormone or its signaling. Pattern of gene expression is shown in the form of heatmap where red color shows up- and blue color shows down-regulation. ABA, abscisic acid; JA, jasmonic acid; SA, salicylic acid; PR, pathogenesis-related; SAR, systemic acquired resistance.

ABA that positively regulates drought tolerance also plays role in defense against both necrotrophic and biotrophic pathogens. For defense against necrotroph, it interacts with SA and JA or ET biosynthesis and signalling^49–53^ (Fig. 4a). ABA has been shown to repress SA induction, the systemic acquired resistance (SAR) pathway and inhibit the accumulation of defense compounds, phenylpropanoids (Fig. 4a)^54–56^. It positively provides resistance against pathogens invading through stomata, such as *P. syringae* ^57^. It also manipulates other pre-invasion defense mechanisms like callose deposition^58–60^. On the other hand, pathogen induced SA also interferes with abiotic stress signaling and lead to drought susceptibility^55, 61^. ABA has been shown to antagonize JA and ET defense signaling, as shown by the irreversible repression of defense genes such as *PDF1.2* ^62^. Thus, crosstalk and convergence of mechanisms in hormone biosynthesis and signalling pathways hint towards possible interaction between drought and pathogen stress over plant interface and modulate consequent abiotic or biotic stress responses. However, the scenario described here varies with different kind of pathogens^24, 62, 63^ which can variably influence the outcome of stress interactions under combined stress. Also, recent molecular studies suggest that specific combined stress invokes novel responses in plants and therefore, those cannot be predicted from the study of either stress individually^6, 12, 64–66^.

In present study, under combined DH stress we observed no change in endogenous concentration of ABA and consistently transcript expression of biosynthesis related genes were not up regulated (Fig. 2; Supplementary Figs S9 and S11). However, negative regulators of ABA signaling were up-regulated (Supplementary Fig. S9). This was in contrast to the activated biosynthesis and signaling of ABA under individual D or H stress (Figs 2, 4 and Supplementary Fig. S11). Unlike ABA, both SA and JA biosynthesis and signaling was greatly enhanced in combined stress over D or H stress (Fig. 2; Supplementary Figs S9 and 11). This consistently resulted in activation of ABI1/2 which negatively regulates ABA signaling (Fig. 4b). *OST1* and *SLAC1* genes involved in ABA-mediated stomatal closure that also required for intact SA signaling were up-regulated in combined stressed (DH) over drought stressed plants (Fig. 4b). Earlier evidences show that absence of ABA increases callose deposition^67^ and phenyl-propanoids. These indicate combined stressed plants converge drought and Pst DC3000 response through enhanced stomatal closure, callose deposition and phenyl-propanoids (Fig. 4b). Thus an upsurge in SA and JA concentrations while unaltered levels of ABA, potentially strengthen signaling events for combined stress tolerance (Fig. 4b). With the absence of ABA at 8 hpt in DH plants, it is also clear that the tolerance to combined drought and pathogen stress is mediated by ABA at early stage of this stress. These results are in sync with the independent abiotic and biotic stress resistance crosstalk where ABA and SA signaling pathways are predominantly antagonistic^55, 68^. Thus, in conclusion the initial suppression of ABA and induction of SA and JA levels at later time points coupled with activated ABA and JA mediated signaling along with suppressed SA mediated signaling could have instigated the robust defense that resulted in reduced bacterial multiplication in combined DH stressed plants. Our results are in line with the observations made by Mohr and Cahil^24^ suggesting the positive role of ABA in imparting susceptibility to *A. thaliana* plants to *P. syringae*.

Plant responses to combined stress can be affected by order of occurrence of stressor^6, 8^. With this purview, we studied hormonal regulation under HD combined stress and noted increased accumulation of ABA, SA and JA at early stress (Supplementary Figs S11 and S12). This was accompanied with up-regulation in ABA, SA and JA signaling. These results indicate a scenario different from the pathways proposed using information from individual stress studies. Consistent with our study, earlier in combined stressed plants, infection with viruses improved tolerance to drought with increase in SA levels^69^. Further, during incompatible interactions, plants invoke robust defense responses which provide a conducive system to study plant defense responses under combined stress. Besides, understanding plant responses towards combined drought and pathogen stress is obstructed by the pathogen inflicted alteration of phytohormones^14^. In this regard, DNH stressed plants displayed unaltered ABA levels in comparison to the individual NH treatment or control, SA and JA-Ile levels however accumulated but were less or similar over NH (Supplementary Figs S11 and S12). DRH treatment lead to significant reduction in ABA levels with no change in SA and JA during early hours of stress and thus depicted the activated defenses (Supplementary Figs S11 and S12).

Taken together, un-induced levels of ABA in DH and DNH plants but activated levels in HD plants at early hours of combined stress treatments suggest that ABA deficiency can increase the pathogen resistance under combined stress (Supplementary Figs S11–S13). Transcriptomic data suggest that regulation of hormone in part occurs at the biosynthetic level (Supplementary Fig. S12). As data based on individual stress study does not take into account the contrasting nature of two stressors and the resultant signaling, these datasets may lead to erroneous conclusions about key regulator under combined stress. This study emphasizes the notion that the complex network of interactions mediates highly specific response of plants to combination of environmental stresses. The response of plant to each combined stress is different and thus it depends on the nature of the pathogen, and time of occurrence of each stressor. Henceforth, the absence of ABA possibly played a key role in combined stress responses through regulation of SA and JA mediated defense pathways.

## Materials and Methods

### Plant growth

*Arabidopsis thaliana* accession Columbia-0 (Arabidopsis Biological Resource Center, accession number CS70000) seeds were sown in pre-weighed agropeat (Prakruthi Agro Tech, Karnataka, India) and vermiculite (Keltech Energies Ltd, Maharashtra, India) mix (3:1 vol/vol) and were stratified at 4 °C for 2 days under dark conditions. Plants were grown at 20 °C constant temperature in growth chamber (PGR15, Conviron, Winnipeg, Canada) under short-day conditions (8 h of light, 16 h of dark) and controlled light intensity of 200 µE m^−2s−1^ and 75% relative humidity. Irrigation was alternated with water and 0.5X Hoagland nutrient solution (Cat # TS1094, Himedia Laboratories, Mumbai, India) every day till the stress treatments commenced.

### Bacterial strains, growth and preparation of inoculum

*Pseudomonas syringae* pv. tomato DC3000 (Pst DC3000), a host pathogen and *P. syringae pv. tabaci* (Psta), a non-host pathogen of *A. thaliana* were used in this study. Both the bacterial strains were grown in King’s B (liquid) medium (Cat # M1544, Hi-media Laboratories, Mumbai, India) supplemented with rifampicin (50 μg/mL) for Pst DC3000. Bacterial cultures were grown at 28 °C with a continuous shaking of 150 rotations per minute till the optical density at 600 nm (OD_600_) reached 0.4. Accordingly, single colony for each of Pst DC3000 and Psta was grown for 12 h. The cultures were centrifuged at 4270 g for 10 min. Resultant pellet was washed thrice with sterile water and was suspended in water. The desired concentration of 5 × 10^3^ colony forming units (CFU)/mL for Pst DC3000 and 1 × 10^5^ CFU/mL for Psta were used to inoculate the leaves (37-d-old plants).

### Stress imposition

#### Individual stress treatments

Individual drought stress (D) treatment was initiated in 32-d old *A. thaliana* plants (Supplementary Fig. S1). Gravimetric method was used to monitor and maintain drought stress level^70^. Plants were grown in saturated soil (10 g dried potting mix; at 100% soil moisture content) at 100% field capacity (FC, Ψw = −2.89 MPa) and irrigation was stopped on 32nd day after germination. Potted plants reached 40% FC five days after water withholding and thereafter drought stress levels were maintained constantly at 40% FC (Ψw = −3.9 MPa) for 24 hours post treatment (hpt) by irrigating the amount of water lost through evapo-transpiration. Gravimetric measurements showed that plants conceived progressive drought stress of 80% and 60% FC for 1 and 2 days respectively before reaching to a final drought stress level of 40% FC. Well-watered control plants were maintained at 100% FC throughout the experiment. Soil water status was correlated with RWC of drought stressed plants where plants at 40% FC showed ~55% RWC as compared to the 90% RWC in turgid leaves of control plants at 100% FC. When compared to control plants, pathogen infiltration did not lead to change in leaf relative water content (RWC) and DRP stressed leaves showed 74.6% RWC. Individual pathogen stress was imposed by infiltrating host (H, Pst DC3000) at 5 × 10^3^ or non-host (NH, Psta) pathogen at 1 × 10^5^ CFU/mL through abaxial side of leaves (37-d-old plants) using needle-less syringe. All the leaves of the plant were infiltrated. These plants were maintained at 100% FC.

### Combined stress imposition

#### Bacterial inoculation on drought stressed plants

Per the combined stress protocol, plants were exposed to drought stress followed by bacterial inoculation i.e., either host (drought host pathogen treatment, DH) or non-host (drought non-host pathogen treatment, DNH) bacteria. Correspondingly, plants were allowed to experience progressive drought stress by stopping irrigation and the above mentioned concentrations of respective bacteria (host pathogen, H and non-host pathogen, NH) were inoculated at 40% field capacity (FC). Plants were maintained at constant drought level (40% FC) along with continued *in vivo* bacterial multiplication for 24 h (Supplementary Fig. S1).

#### Drought stress imposition on pathogen inoculated plants

This protocol of combined stress involved only host pathogen along with drought stress (host pathogen and drought, HD). Leaves of 32-d-old *A. thaliana* (grown under fully saturated conditions, at 100% FC) were inoculated with host pathogen and thereafter subjected to water withdrawal. Thus, at the time of sample harvest, these plants had continued *in planta* bacterial multiplication for 5 days and also experienced 40% FC drought stress for 1 day. The corresponding individual stress involved inoculation of host pathogen on 32-d-old plant maintained at 100% FC throughout the experimental duration (host pathogen 5 days, H5) (Supplementary Fig. S1).

#### Pathogen inoculation on drought stress recovering plants

A batch of drought stressed plants was infiltrated with host pathogen during recovery (drought recovery host pathogen, DRH). In this protocol, potted plants were initially exposed to drought stress and once the soil moisture level went down to 40% FC, plants were re-watered for 2 h and immediately thereafter host-pathogen at the said concentration was inoculated. These plants recovering from drought stress coupled with progressive pathogen multiplication were maintained at 100% FC till the completion of the experiment (24 hpt) (Supplementary Fig. S1).

#### Sample harvest

Leaf samples were taken at 2, 8 and 24 hours post treatment. Samples for each time point were collected from different set of plants. Harvested leaves were immediately quenched into liquid Nitrogen. Biological replicates from at least five plants per treatment were considered.

#### Phytohormone profiling

The frozen samples were lyophilized at ultra-low temperature (Cat # 7934030, Labconco FreeZone 6^+^ Freeze Dry System, Missouri, USA) and precise weight of each lyophilized sample was taken. The lyophilized tissue was vortexed to fine powder with Zirconium beads at ultra-low temperature. Following protocol described in Tsukahara *et al*.^71^, solid phase extraction and LC-MS analysis were carried out. Briefly, a mix of internal standard [D_6_-ABA, D_4_-SA, D_2_-GA_4_, D_5_-tZ, D_6_-iP (OlChemim, Czech Republic), D_2_-JA (Tokyo Kasei, Japan), ^13^C_6_-JA-Ile (kindly gifted by Dr. Yusuke Jikumaru, Riken, Japan; present affiliation: Agilent Technologies Japan, Ltd), and D_2_-IAA (CDN Isotopes, Canada)] and extraction solvent [1% acetic acid (AcOH), 80% acetonitrile (MeCN)] was added to the lyophilized powdered tissue, incubated for 1 hour at 4 °C and centrifuged at 3000 g for 10 min at 4 °C. The pellet was rinsed with extraction solvent again and centrifuged at 3000 g for 10 min at 4 °C. The two supernatants were combined and evaporated to water containing 1% AcOH in a vacuum evaporator. The extracted sample was loaded onto pre-wet Oasis HLB 1 cc extraction cartridge (Waters Corporation, Milford, MA, USA). After washing with 1% AcOH, hormones were eluted with 1% AcOH, 80% MeCN. The eluate was evaporated to water containing 1% AcOH using centrifugal vacuum evaporator and loaded onto pre-wet Oasis MCX cartridge (Waters Corporation, Milford, MA, USA). After washing with 1% AcOH, the acidic fraction was eluted with 1% AcOH, 80% MeCN. A portion of the acidic fraction was evaporated to dryness and reconstituted in 1% AcOH for analysis of SA. The MCX cartridge was further washed with 5% aqueous ammonia, and the basic fraction was eluted with 40% MeCN containing 5% ammonia. The basic fraction was evaporated to dryness and reconstituted in 1% AcOH for analysis of tZ and iP. The remaining acidic fraction was evaporated to water containing 1% AcOH and loaded onto pre-wet Oasis WAX 1-cc extraction cartridge (Waters Corporation). The cartridge was washed with 1% AcOH and the remaining hormones were eluted with 1% AcOH, 80% MeCN. The eluate was evaporated to dryness and reconstituted in 1% AcOH and subjected to analysis of ABA, IAA, GA_4_, JA, and JA-Ile. Phytohormone quantification was performed by Agilent 1260–6410 Triple Quad LC/MS (Agilent Technologies Inc., Santa Clara, CA, USA) equipped with a ZORBAX Eclipse XDB-C18 column and XDB-C8 Guard column (Agilent Technologies Inc.).

The conditions of liquid chromatography and parameters of LC-ESI-MS/MS analysis for each hormone are described in Table 1. The mass chromatograms obtained from LC-ESI-MS/MS analysis of internal standards and samples are presented in Supplementary Fig. S3.

**Table 1:** Details of parameters used for LC-ESI-MS/MS analysis of abscisic acid (ABA), jasmonic acid (JA), jasmonoyl-isoleucine (JA-Ile) and salicylic acid (SA), indoleacetic acid (IAA), *trans*-zeatin (tZ), isopentenyladenine (iP) and gibberellin A_4_ (GA_4_).

Hormone	LC method				Retention time (min)	ESI mode	MS/MS transitions (m/z)	Time segment (min)	Collision energy (V)	Fragmentor voltage (V)
	Solvent A	Solvent B	Gradient (Composition of solvent B)	Flow rate (ml/min)						
IAA	Water + 0.01% (v/v) acetic acid	Acetonitrile + 0.05% (v/v) acetic acid	3 to 50% in 20 min	0.4	9.8	Positive	176/130	9.2–11.8	10	90
D_2_-IAA							178/130			
ABA					12.6	Negative	263/153	11.8–13.5	5	130
D_6_-ABA							269/159			
JA					14.4	Negative	209/59	13.5–15.5	15	135
D_2_-JA							211/59			
GA_4_					16.8	Negative	331/257	15.5–17.3	18	160
D_2_-GA_4_							333/259			
JA-Ile					18.0	Negative	321/130	17.3–20.0	14	140
^13^C_6_-JA-Ile							338/136			
SA	Water + 0.1% (v/v) formic acid	Acetonitrile + 0.1% (v/v) formic acid	3 to 98% in 10 min	0.4	5.6	Negative	137/93	2.0–10.0	12	90
D_4_-SA							141/97			
tZ	Water + 0.01% (v/v) acetic acid	Methanol + 0.2% (v/v) acetic acid	3 to 97% in 16 min	0.25	8.4	Positive	220/136	5.0–11.0	8	100
D_5_-tZ							225/137,137*			
iP					12.6	Positive	204/136	11.0–16.0	18	110
D_6_-iP							210/137			

*Two fragment ions.

#### Data collection and statistical analysis

Data for a minimum 5 biological replicates was collected at designated time points. Outliers were removed using boxplot (the values beyond 1.5 IQR, inter-quartile range were removed)^72^. Data acquired under D treatment was normalized with well-watered (absolute control) and H, NH, H5, DH, DNH, HD and DRH treatment with mock (water only) infiltrated plants. Fold change in hormone levels in treatment samples was calculated over respective controls. Data presented are average of at least three biological replicates. Error bars represent ± SEM. Statistical significance in different treatments over respective controls (drought over absolute control and pathogen or combined stress over mock control) was obtained by student’s t-test at p value < 0.05. Statistical significance among different treatments were obtained by one-way analysis of variance (ANOVA) and comparisons among different treatments were done by applying the least-significant difference post-hoc Tukey’s test (p < 0.05) (SigmaPlot 11.0, Systat Software Inc., California, USA). Raw data, details of numbers of biological replicates and standard error of means are mentioned in the Supplementary File S1.

#### Quantitative Real Time PCR

Leaf sample (100 mg fresh weight) from third tier of rosette was harvested at 24 hpt and was used for isolation of total RNA employing Trizol reagent (Cat # 74904, Qiagen, Hilden, Germany) as per manufacturer’s instructions. RNA was quantified using NanoDrop ND-1000 spectrophotometer (Thermo Scientific, Massachusetts, USA). First strand cDNA was synthesized from 5 μg of total RNA in a reaction volume of 50 μL using Verso™ cDNA synthesis kit (Cat # AB1453A, Thermo Scientific, Massachusetts, USA). Primers specific to target genes were designed using NCBI primer designing tool (https://www.ncbi.nlm.nih.gov/tools/primer-blast/). Primer details are listed in Supplementary Table S2. Transcript expression of selected genes was quantified by real-time PCR (RT-qPCR) using ABI Prism 7000 sequence detection system (Applied Biosystems, California, USA). Template cDNA was (1 µL) was mixed with 750 nM of the gene specific primers and 5.0 µL SYBR Green PCR master mix (Applied Biosystems, USA) in a final volume of 10 µL. Ct values obtained for *AtACTIN2* (AT3G18780) gene was used to normalize target gene expression data. Relative gene expression in treatment samples was quantified using comparative D cycle threshold (CT) method over respective control samples^73^. For all the RT-qPCR experiments, four independent biological replicates were performed. Samples were harvested from a batch of plants maintained under same conditions beside those used for phytohormone profiling.

## Electronic supplementary material

Supplementary information

Supplementary File S1

Supplementary File S2

Supplementary File S3

Supplementary file S4
