# Supplementary material for: Global profiling of phytohormone dynamics during combined drought and pathogen stress in Arabidopsis thaliana reveals ABA and JA as major regulators
Source: Sci Rep. 2017 Jun 21;7:4017. doi: 10.1038/s41598-017-03907-2 (PMC5479852; doi:10.1038/s41598-017-03907-2)
Supplement: Supplementary file 1 — Supplementary information [file 41598_2017_3907_MOESM1_ESM.pdf]

Supplementary figures 1-13
Supplementary tables 1 & 2

Global profiling of phytohormone dynamics during combined drought and pathogen stress in Arabidopsis reveals ABA and JA as major regulators

Aarti Gupta[1], Hiroshi Hisano[2], Yuko Hojo[2], Takakazu Matsuura[2], Yoko Ikeda[2], Izumi C. Mori[2], Muthappa Senthil-Kumar[1] *

[1]National Institute of Plant Genome Research, Aruna Asaf Ali Marg, JNU campus, New Delhi 110067 India

[2]Institute of Plant Science and Resources, Okayama University, Kurashiki, 710-0046, Japan

DOI:10.1038/s41598-017-03907-2

Supplementary Figure S1

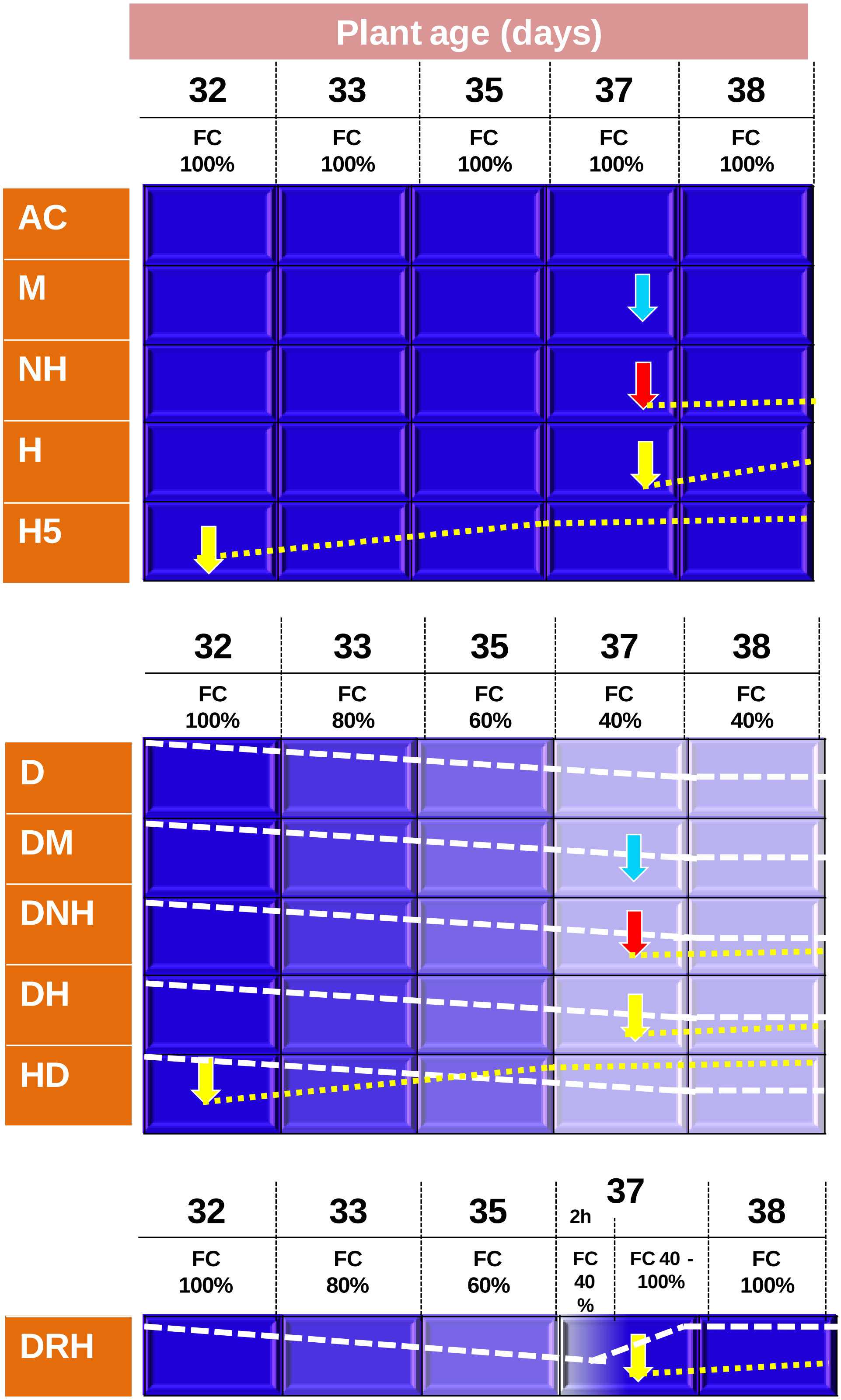

Supplementary Figure S1: Protocol for imposition of different stress treatments. *Arabidopsis thaliana* was grown in controlled environmental conditions with 8 h light/16 h dark photoperiod, 20 ˚C temperature and 70% relative humidity. Plants at 32-d-old stage, were subjected to stress treatments. Drought stress was applied such that soil moisture content was at 40% field capacity, FC; equal to Ψw -3.9 Mpa. For host pathogen; *Pseudomonas syringae* pv. tomato DC3000 (Pst DC3000) and non host pathogen; *P. syringae* pv. tabaci (Psta) treatments, inoculum concentrations of 5 X 10^3 CFU/mL and $1X10^5$ CFU/mL respectively were syringe infiltrated. The effective concurrent stress was subjected for 1 day. Different set of plants were maintained as AC- well watered with no inoculation (absolute control); D- drought at 40 % FC; M- well watered plants were water infiltrated at 37 d; H-well watered plants were inoculated with Pst DC3000 at 37 d; NH-well watered plants were inoculated with Psta at 37 d; DNH- Drought stressed plants were inoculated with non-host pathogen at 37 d; DH- Drought stressed plants were inoculated with host pathogen at 37 d and were maintained for 1 d under concurrent stress; HD- 32 d old plants were inoculated with Pst DC3000 followed by water withdrawal till plant reaches 40% FC and experienced progressive pathogen and drought concurrent stress; H5 – on first day of experiment Pst DC3000 was inoculated on plants continuously maintained under 100% FC; DRH represents pathogen after drought recovery wherein plants at 40% FC were well watered for 2 h on 37th day and thereafter pathogen was inoculated. Pots at FC 40% were maintained at the respective FC, by replenishing the lost amount of water, till the end of the experiment. Leaf samples were collected at 2, 8 and 24 hours post treatment (hpt). Soil moisture level is depicted as the shade of blue color, the blue arrow shows mock infiltration with water, yellow arrow indicates host pathogen infiltration, red arrow denotes infiltration with non-host pathogen and dotted lines represent *in planta* pathogen multiplication. Dotted yellow line indicates presumed bacterial multiplication and dotted while line indicate presumed reduction in field capacity.

Supplementary Figure S2

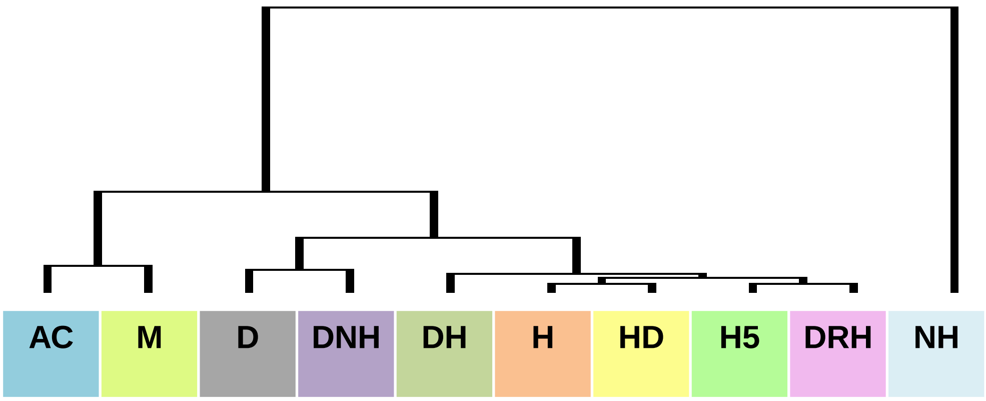

Supplementary Figure S2: Illustration of hierarchical clustering of phytohormone profiles from individual and combined stressed *A. thaliana*. In order to show the relatedness of different treatments, condition based clustering with Pearson's correlation was performed using average hormone concentration values at 24 hpt under different conditions including absolute control (AC), mock (M), drought (D), host-pathogen (H), non-host pathogen (NH), host pathogen stress for 5 days (H5), combined drought followed by host pathogen (DH), combined host pathogen with drought (HD), combined drought and non-host pathogen (DNH) and combined drought-recovery-pathogen treatment (DRH) and is represented here. The result clearly indicates that the treatments involving pathogen inoculation under watered conditions (H, H5, DRH) were more related, while the drought stressed treatments associated close together.

Supplementary Figure S3

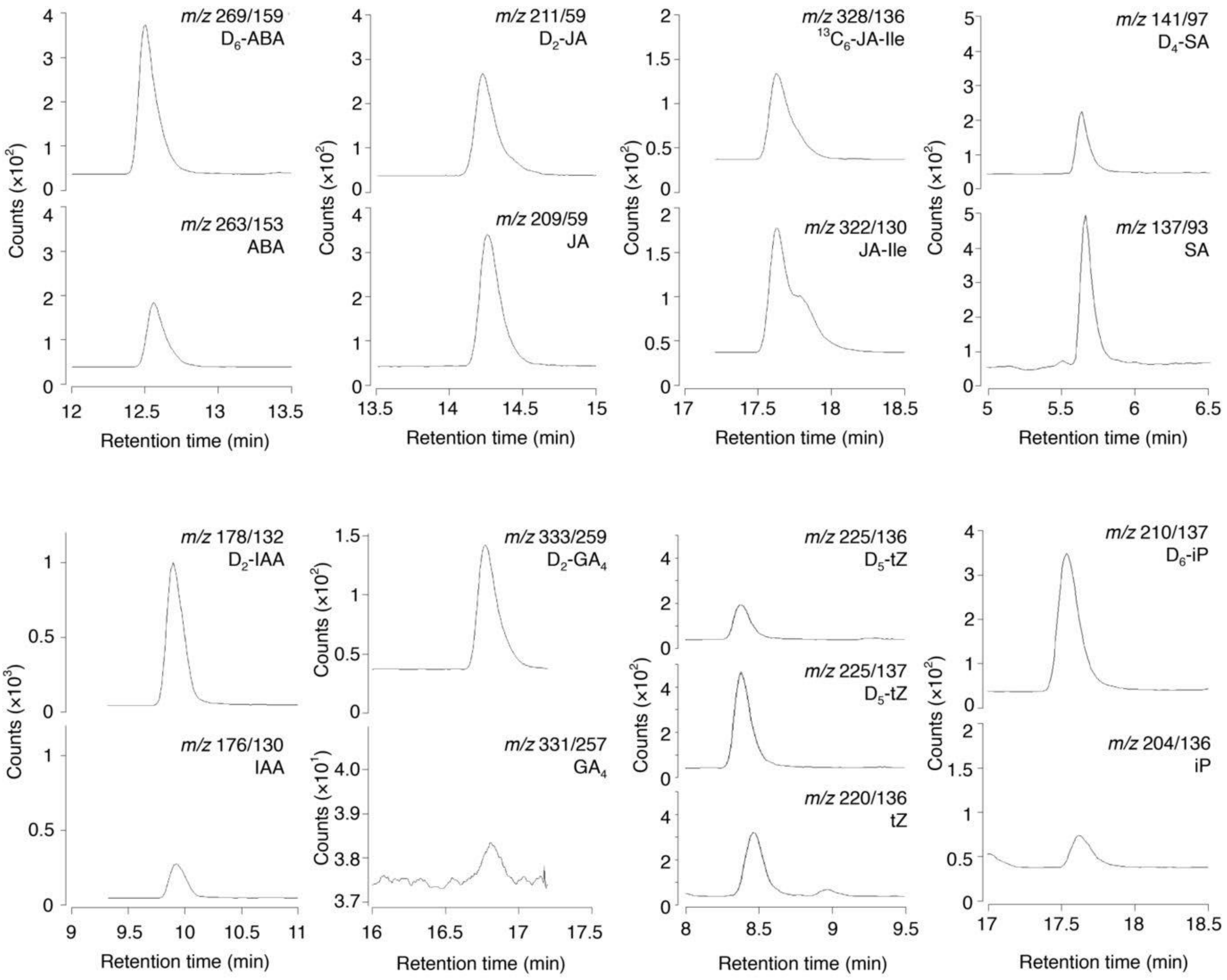

Supplementary Figure S3. Representative chromatograms of phytohormones' standards and of those obtained from control *A. thaliana*. Typical chromatograms and multiple-reaction-monitoring mass transitions of abscisic acid (ABA), jasmonic acid (JA), jasmonoyl isoleucine (JA-Ile), salicylic acid (SA), indoleacetic acid (IAA), gibberellin A_4 (GA_4), *trans*-zeatin (tZ), isopentenyladenine (iP) and corresponding internal standards analyzed by liquid chromatography-triple quadrupole mass spectrometry in control *Arabidopsis thaliana* are shown. JA-Ile consists of stereoisomers possessing different retention times as observed as the shoulder peak. D_5-tZ generates two fragment ions due to distribution of deutrium in the fragment ion.

Supplementary Figure S4

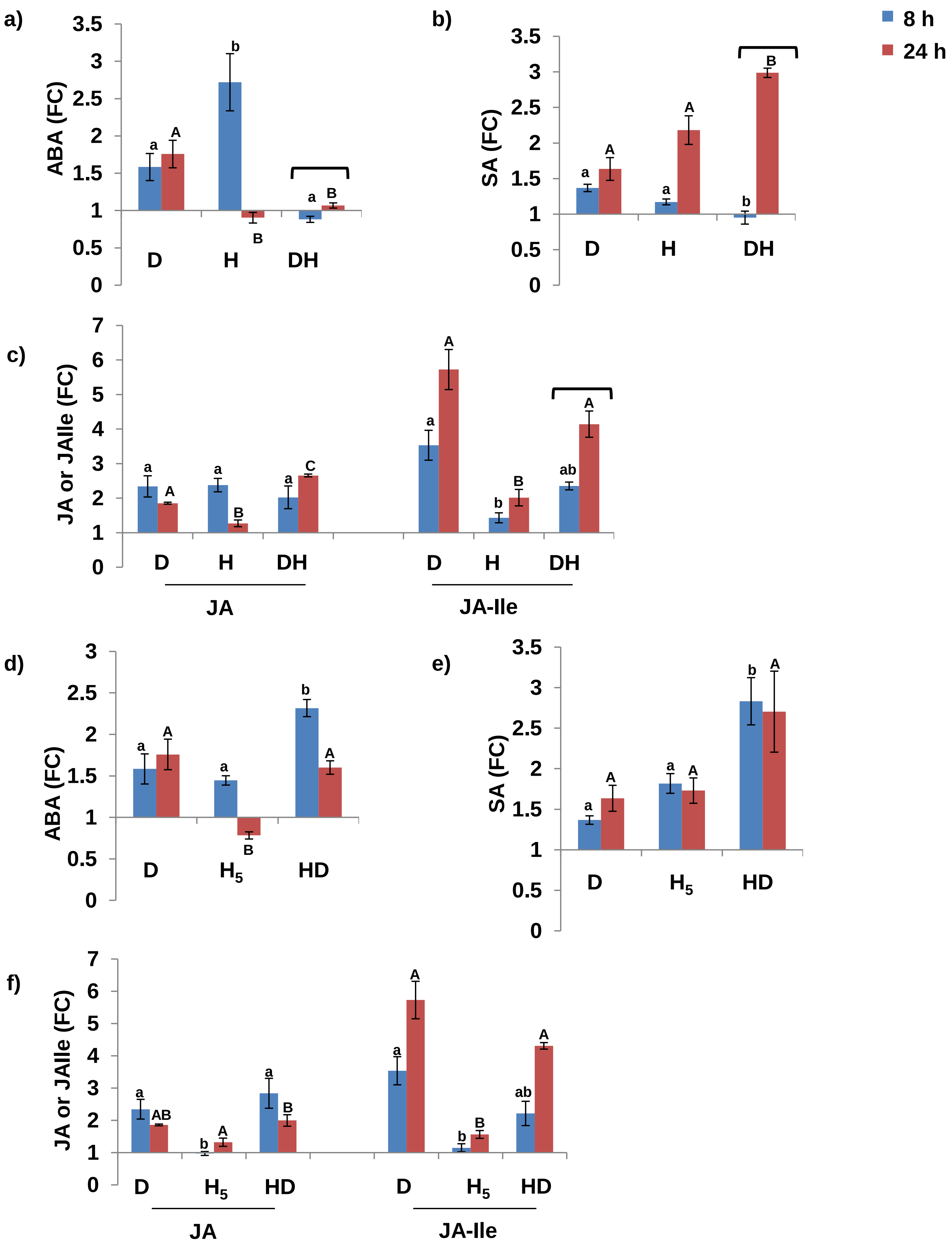

Supplementary Figure S4. Phytohormone levels in leaves of *A. thaliana* exposed to individual host, drought and their combined treatments. Different phytohormone levels at 8 and 24 hours post treatment (hpt) are represented in the form of fold change in drought (D), host pathogen (H, *Pseudomonas syringae* pv. tomato DC3000), (H5, pathogen infection for 5 days), combined drought stress followed by host pathogen (DH) and combined host pathogen followed by drought stress (HD) over their respective controls. Plants were subjected to individual drought and host pathogen stresses and their combination and all leaves were used to quantify hormone concentrations. Fold change in hormone levels in individual drought stressed samples were assessed over absolute control. Fold change in hormone levels under host pathogen infection (H) and combined stress (DH) was calculated over mock inoculated samples and is presented for ABA **(a)**, SA **(b)** and JA/JA-Ile **(c)**. Fold change in hormone levels under prolonged host pathogen infection (H5) and combined stress (HD) was calculated over mock inoculated samples and is presented for ABA **(d)**, SA **(e)** and JA/JA-Ile **(f)**. Each data point represents the mean of at least three independent replicates and error bars denote standard error of mean. Significant differences among different treatments was analyzed by ANOVA and posthoc Tukey's test where different letters denote significance among means. Small letters represent comparison among treatments at 8 h and capital letters represent comparison at 24 h time point. Values for drought stress were from the same experiment as presented in Fig. 1. Raw values for phytohormone concentrations and calculated standard error of mean over respective controls are presented in Supplementary File S1.

Supplementary Figure S5

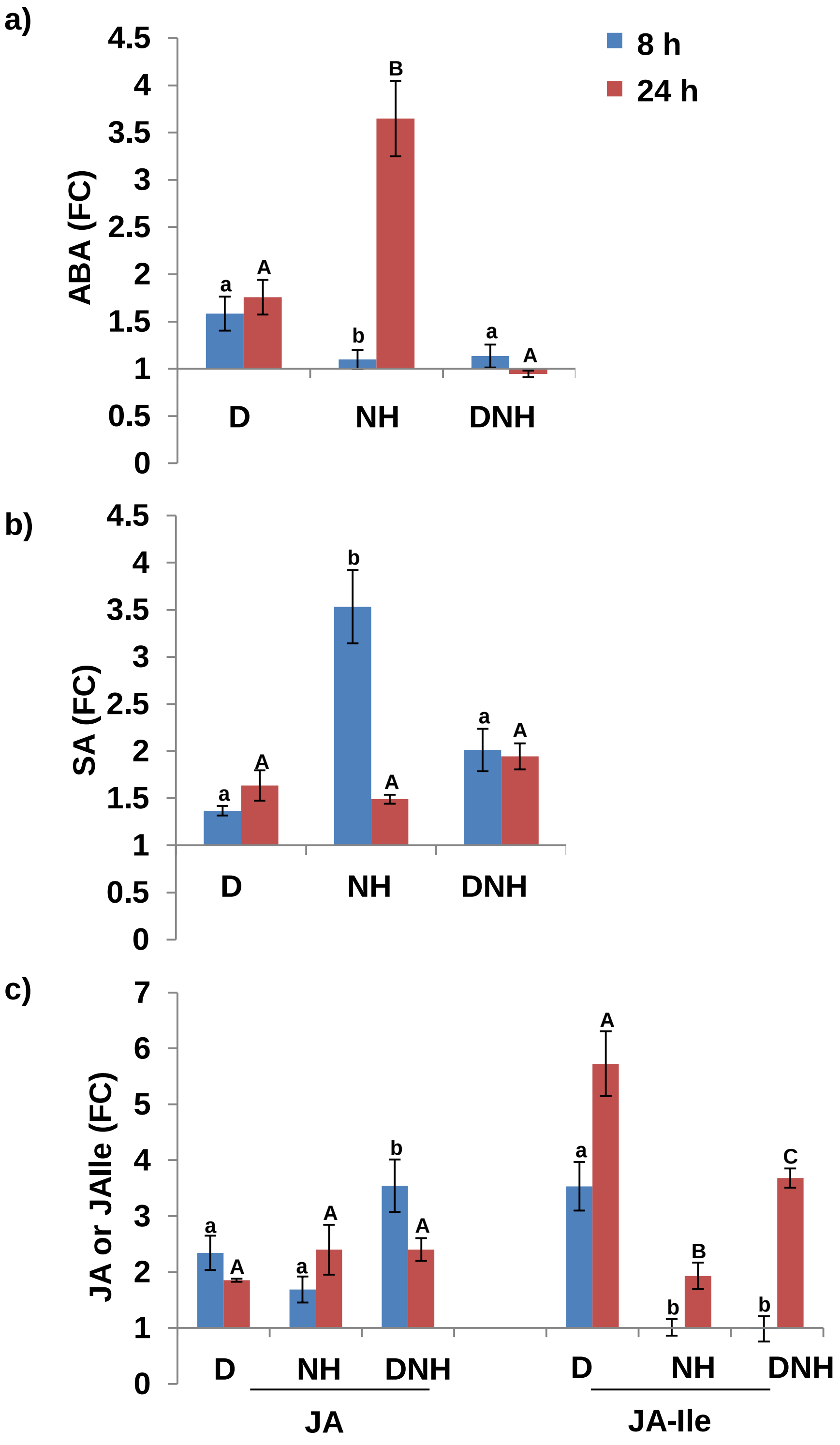

Supplementary Figure S5. Relative phytohormone profile of *A. thaliana* exposed to individual drought, non-host pathogen and their combined treatments. Plants were subjected to individual drought (D) and non-host pathogen (NH, *Pseudomonas syringae* pv. tabaci) treatments and combined drought followed by non-host pathogen stress (DNH). Treated leaves were used to quantify hormone concentrations. Different phytohormone levels at 8 and 24 hours after post individual and combined treatments (hpt) are represented in the form of fold change over their respective controls. Fold change in hormone levels in individual drought stressed samples were assessed over absolute control. Fold change in hormone levels under non-host pathogen inoculation and combined treatments was calculated over mock inoculated samples and is presented for ABA **(a)**, SA **(b)** and JA/JA-Ile **(c)**. Each data point represents the mean of at least three independent replicates and error bars denote standard error of mean. Significant differences among different treatments was analyzed by ANOVA and posthoc Tukey's test where different letters denote significance among means. Small letters represent comparison among treatments at 8 h and capital letters represent comparison at 24 h time point. Values for drought stress were from the same experiment as presented in Fig. 1. Raw values for phytohormone concentrations and calculated standard error of mean over respective controls are presented in Supplementary File S1.

Supplementary Figure S6

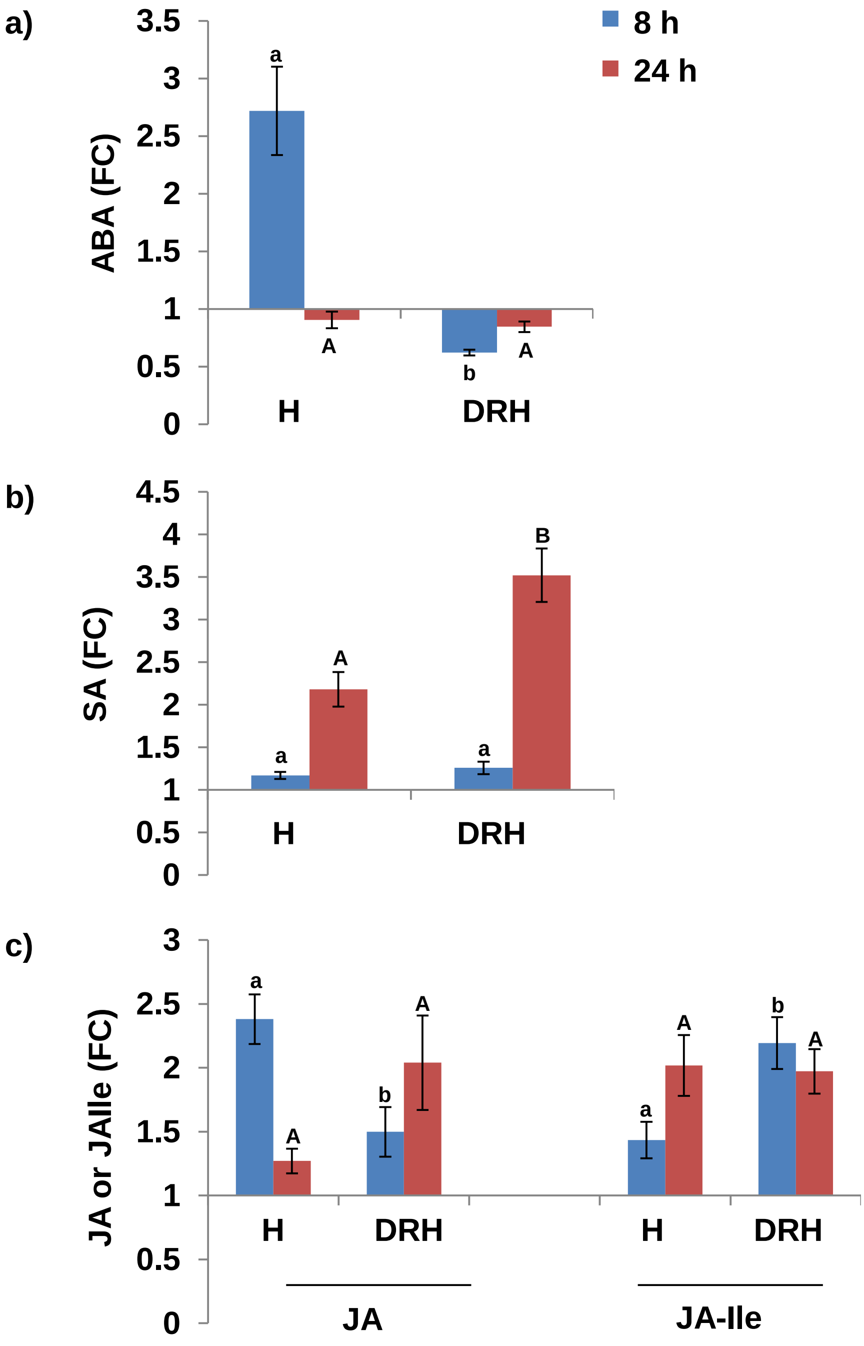

Supplementary Figure S6. Alteration in phytohormone profile in *A. thaliana* exposed to host pathogen infection under well watered and drought-recovery state. Plants were exposed to host pathogen infection under well-watered (H) and drought stress-recovered state (DRH). Treated leaves were used to quantify hormone concentrations. Different phytohormone levels at 8 and 24 hours post treatment (hpt) are represented in the form of fold change over mock inoculated controls and is presented for ABA **(a)**, SA **(b)** and JA/JA-Ile **(c)**. Each data point represents the mean of at least three independent replicates and error bars denote standard error of mean. Significant differences among different treatments was analyzed by ANOVA and posthoc Tukey's test where different letters denote significance among means. Small letters represent comparison among treatments at 8 h and capital letters represent comparison at 24 h time point. Values for drought stress were from the same experiment as presented in Fig. 1. Raw values for phytohormone concentrations and calculated standard error of mean over respective controls are presented in Supplementary File S1.

Supplementary Figure S7

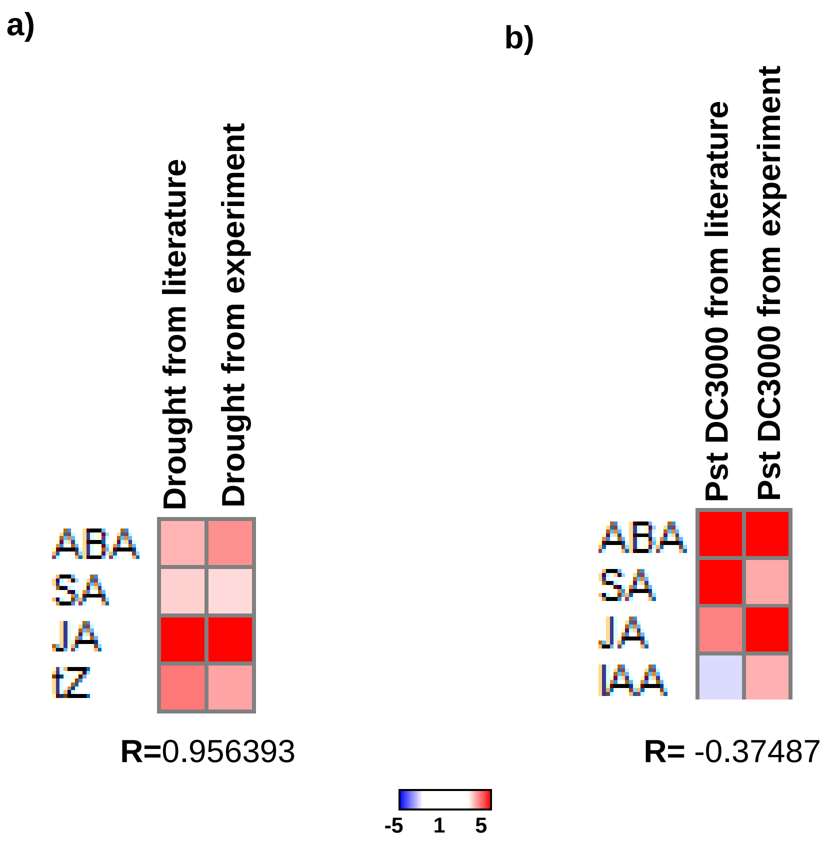

Supplementary Figure S7. Comparison of phytohormone modulations in *A. thaliana* under drought or Pst DC3000 from different sources. Comparative trend in phytohormones values (relative to respective controls) obtained in present study to literature derived data is presented in the form of heatmap. Comparison of phytohormone modulation during drought stress **(a)** and Pst DC3000 **(b)** is represented here. Values were obtained from different studies and are plotted in the form of heatmap. Fold change values obtained from literature under drought stress are represented as drought from literature and under *P. syringae* stress are represented as PstDC3000 from literature. Fold change values observed in present study (recorded at 8 hpt) under drought stress is represented as drought from experiment and under *P. syringae* as Pst DC3000 from experiment. R denotes the Pearson's correlation coefficient. Color scheme with red boxes shows up-regulation and blue boxes represents down-regulation in hormone levels. Results indicate high correlation between the hormone values derived from present study and literature. In case of pathogen stress, though the trend between the values obtained from present study and literature data was similar, the very low R value can be attributed to the experimental variation likely by different levels and method of stress. Details of fold changes, and the referred literature are provided in Supplementary File S2.

Supplementary Figure S8

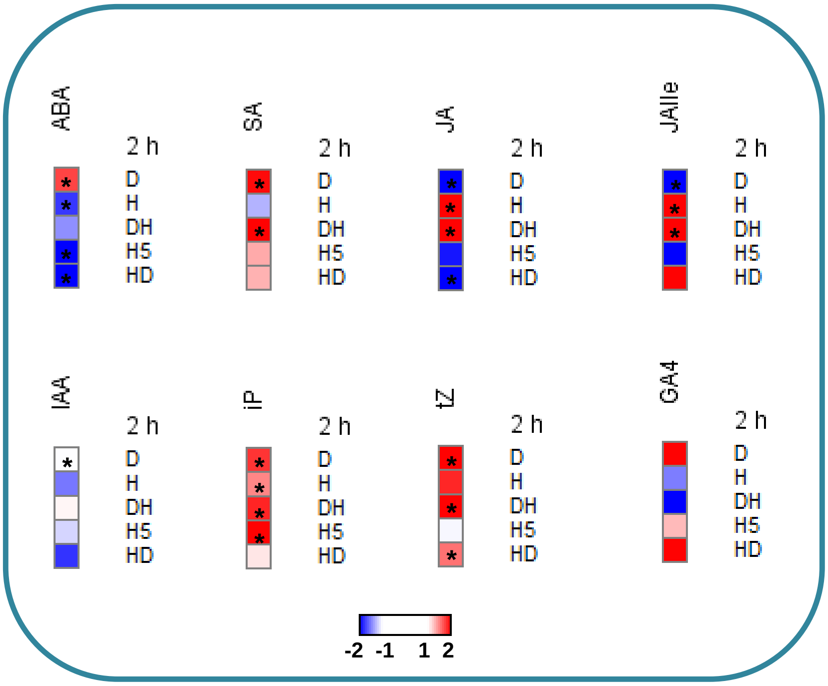

Supplementary Figure S8: Phytohormone profile in *A. thaliana* exposed to individual drought and host pathogen stresses and their combinations. Hormone levels under individual and combined stresses were quantified at 2 hpt and fold change over control was calculated. Fold change values were used to plot heatmap. Color blocks in red and blue indicate up-regulated and down-regulated levels in hormones respectively. * indicates significant change in hormone levels over control at $p \leq 0.05$. Significant difference in treatments over control was calculated using Students' t-test. Raw values with standard error of means and number of replicates are mentioned in supplementary File S1. D; drought, H; host pathogen Pst DC3000, DH; combined drought and host pathogen stress, H5; host pathogen Pst DC3000 infection for 5 days, HD; combined host pathogen and drought stress.

Supplementary Figure S9

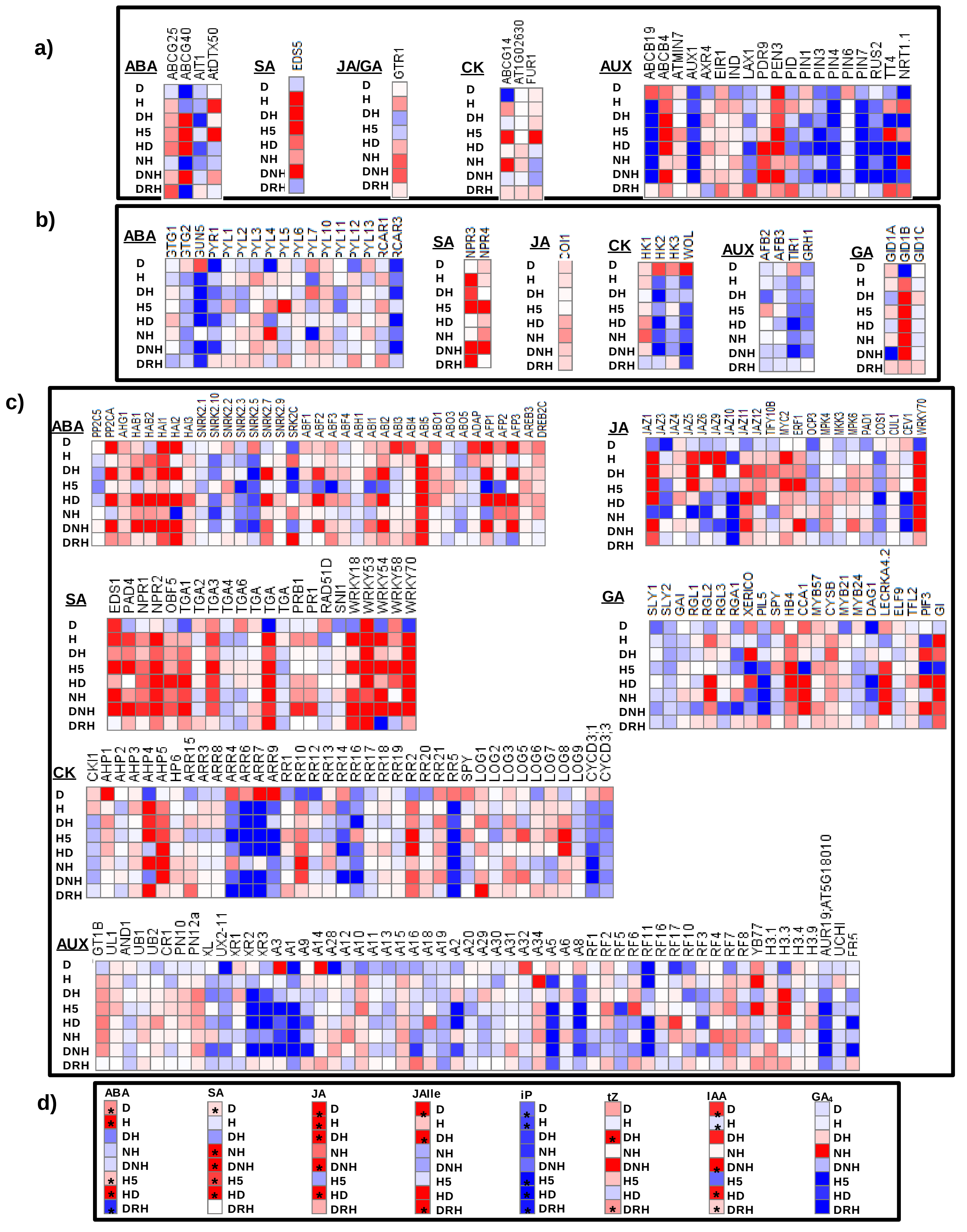

Supplementary Figure S9. Transcriptome changes possibly mediated by phytohormones in *A. thaliana* under individual and combined stresses. Transcriptome analysis for hormone transport**, (a)** perception **(b)** and signaling **(c)** related genes is represented in the form of heatmaps and was correlated with phytohormone levels at 8 hpt represented in the form of heatmaps **(d).** Differentially expressed genes under individual and combined stressed treatments over control were identified from microarray study (GEO NCBI accession no. GSE79681). Genes involved in hormone transport, perception and signaling were identified through manual curation in literature and Arabidopsis Hormone Database 2.0 (http://ahd.cbi.pku.edu.cn/). Full details of genes names and corresponding ID are provided in Supplementary File S2. Significant difference in treatments over control was calculated using Students' t-test. * indicates significant change in hormone levels over control at $p \leq 0.05$. Raw values with standard error of means and number of replicates are mentioned in Supplementary File S1. D; drought, H; host pathogen Pst DC3000, DH; combined drought and host pathogen stress, H5; host pathogen Pst DC3000 infection for 5 days, HD; combined host pathogen and drought stress, NH; non host Psta, DNH; combined drought and non host, DRH; combined drought recovery host pathogen stress.

Supplementary Figure S10

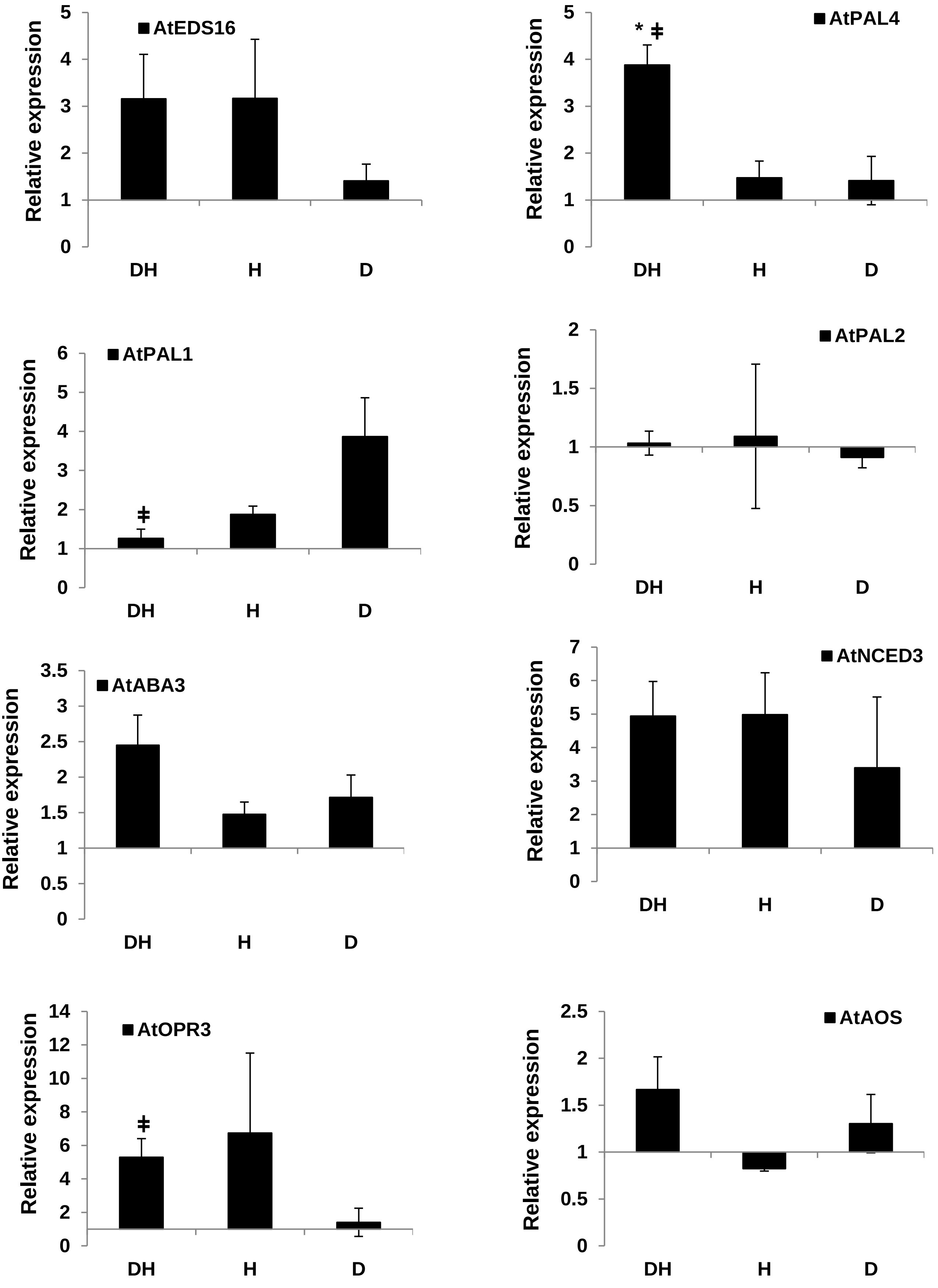

Supplementary Figure S10. RT-qPCR based transcript expression profile of key hormone biosynthetic genes. Transcript expression profile of key genes involved in hormone biosynthesis under individual drought (D), host pathogen (H) and combined drought and host pathogen (DH) stresses was assessed by RT-qPCR. Graph represents relative expression of the genes under individual and combined stress. *AtActin2* was used as reference gene for data normalization. Fold change under DH or H stress was calculated over mock control. Fold change in expression under D stress was calculated over absolute control. Each bar for DH stress represents average of four biological and for D or H stress represents average of three biological replicates ± SEM. * or **‡** denote significance under DH stress over H or D stresses respectively at $P< 0.05$ (Student's *t* test). Expression values of each gene under each stress treatments are provided in Supplementary File S4.

Supplementary Figure S11

DH combined stress

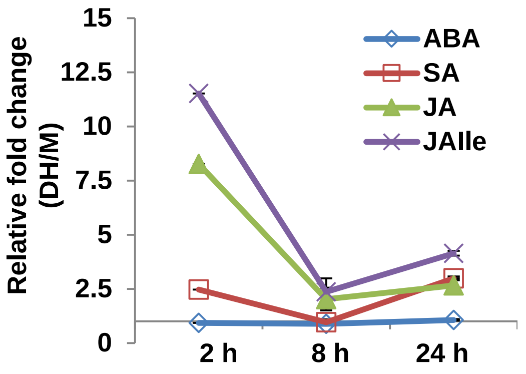

	8 hpt	24 hpt
JA	↑↑	↑↑↑
JA-Ile	↑↑	↑↑↑↑↑↑
SA	=	↑↑↑↑
ABA	=	=

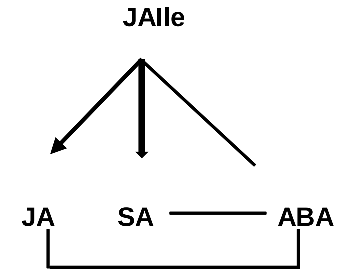

HD combined stress

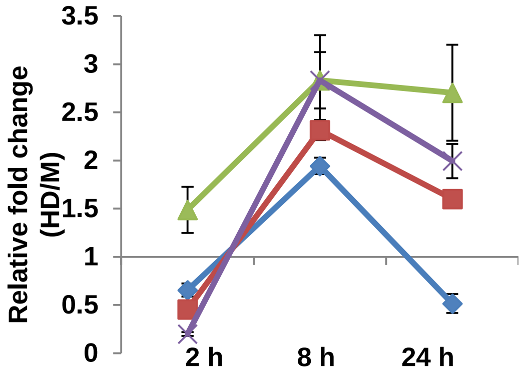

JA	↑↑↑↑	↑↑↑↑
JA-Ile	↑↑↑↑	↑↑
SA	↑↑↑	↑
ABA	↑↑	↓

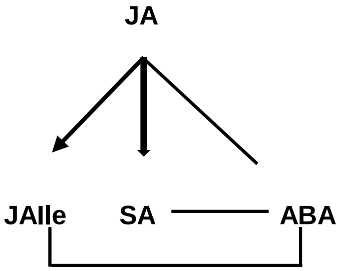

DRH combined stress

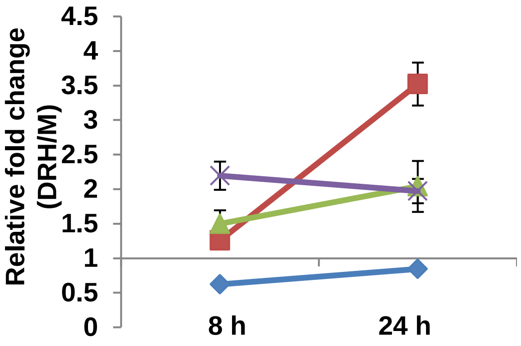

JA	↑	↑↑
JA-Ile	↑↑	↑↑
SA	=	↑↑↑↑↑
ABA	↓	=

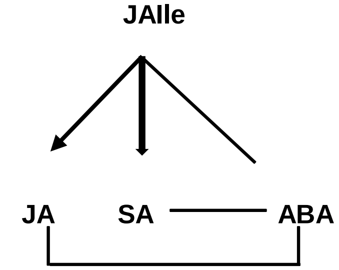

DNH combined stress

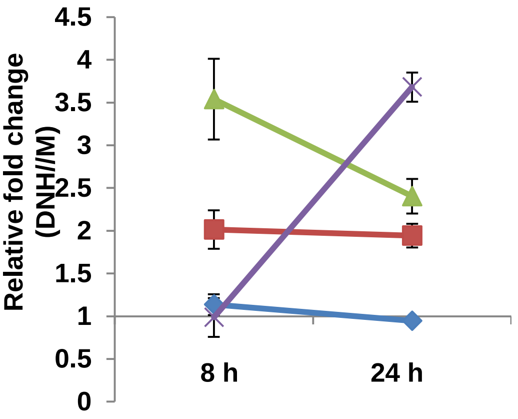

JA	↑↑↑↑↑	↑↑↑
JA-Ile	=	↑↑↑↑↑
SA	↑↑	↑↑
ABA	=	=

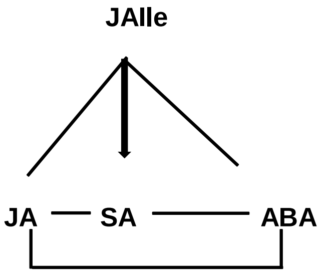

Supplementary Figure S11. Overview of phytohormone modulation under combined stresses based on experimental evidence. Fold change values in JA, JA-Ile, SA and ABA were calculated at 2h, 8h and 24h time point over mock control. Phytohormone concentrations were not determined at 2 h under DRH and DNH combined stress treatments. Models are postulated for each treatment based on the relative fold change obtained in experiment. The phytohormone with highest fold change was placed first in order. Table shows up- or down-regulation in hormone levels at 24 h time point. Results indicate the link between initial increase in JA accompanied by increased SA levels however there was no change in ABA content in treatments involving DH, DRH and DNH stress. This might be a contributory factor to the enhanced defenses leading to reduced pathogen growth under these combined stress scenarios. During HD combined stress however, an initial decrease in JA-Ile, SA and ABA was accompanied with increased JA content. At later time point, the JA, JA-Ile and SA content upsurged and was associated with a dip in ABA content. These observations could link between the two events, the modulation in hormone content and bacterial growth under different combined stresses. DH; combined drought and host pathogen stress, HD; combined host pathogen and drought stress, DRH; combined drought recovery host pathogen stress, DNH; combined drought and non host pathogen stress. Each upward arrow correspond to 0.5 fold accumulation, downward arrow indicates 0.5 fold reduction and equals to sign represent no change in phytohormone concentrations when compared to mock control.

Supplementary Figure S12

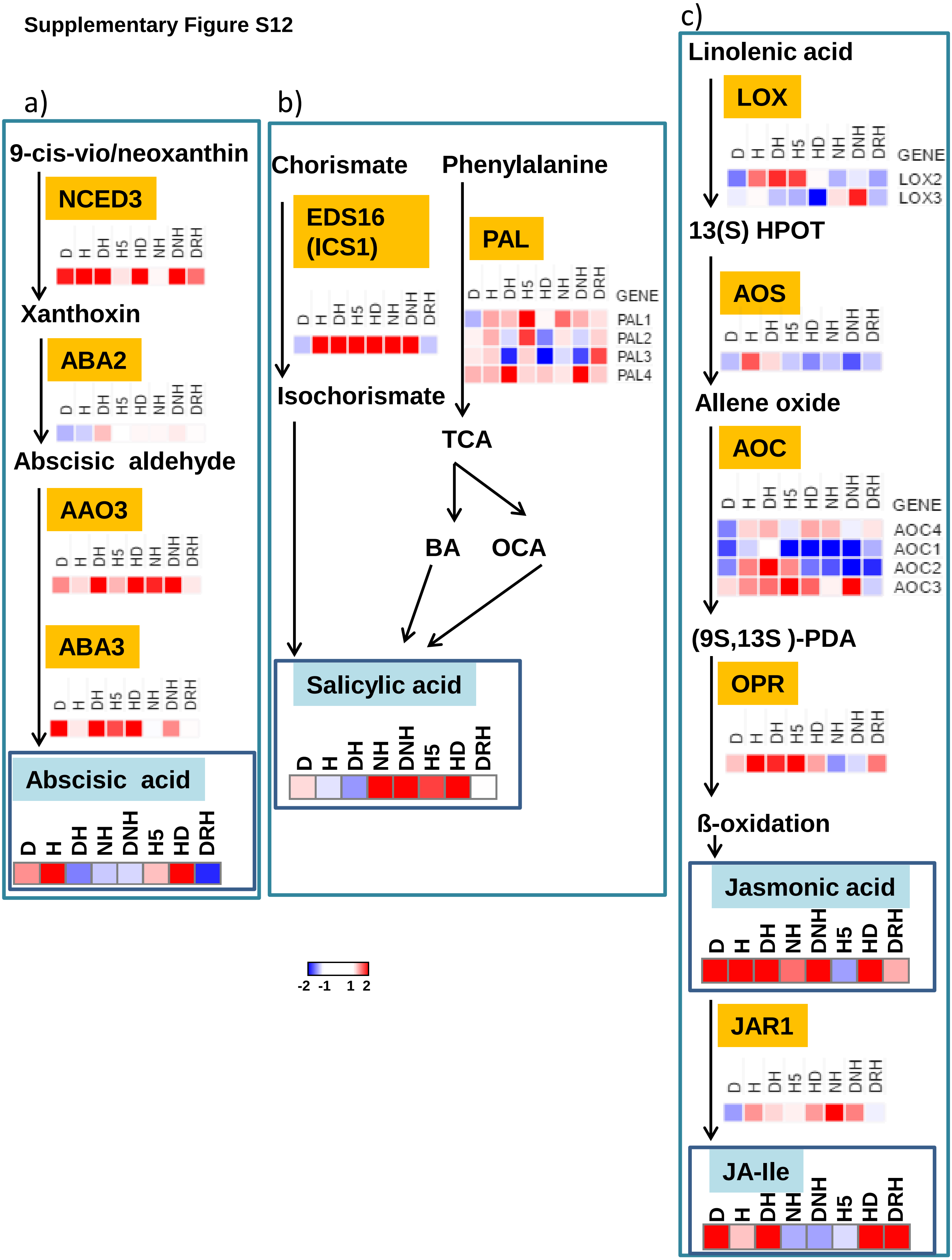

Supplementary Figure S12. Influence of different stresses on hormone biosynthesis pathway. Information on Key steps involved in three major phytohormone; ABA **(a)**, SA **(b)**, and JA **(c)** biosynthesis pathway was retrieved from literature. Microarray data derived expression profile of biosynthesis genes under individual and combined stress was used to plot heatmaps and is presented alongside corresponding step. Relative hormone levels under individual and combined stresses (over their respective controls) at 8hpt is depicted in form of heatmaps and are lined by box. Color boxes in red and blue represent up- and down-regulation in gene expression or hormone levels. Yellow boxes represent enzymes catalyzing corresponding biosynthesis step. NCED3, nine-cis-epoxycarotenoid dioxygenase 3;ABA2, aba deficient 2; AAO3, abscisic aldehyde oxidase 3; ABA3, aba deficient 3; EDS16, enhanced disease susceptibility to ERYSIPHE ORONTII 16 or ICS1, isochorismate synthase 1; PAL, phenylalanine ammonia-lyase; TCA, Trans-Cinnamic acid; BA, Benzoic acid; OCA, O-Coumaric acid; LOX3, lipoxygenase 3; HPOT, hydroperoxy Octadecatrienoic acid; AOS, allene oxode synthase; AOC, allene oxide cyclase 4; (9S,13S)-12-oxo-PDA, (9S,13S)-12-oxo-phytodienoic acid; OPR3, oxophytodienoate-reductase 3. phytohormone levels at 8 hpt represented in the form of heatmaps
.

Supplementary Figure S13

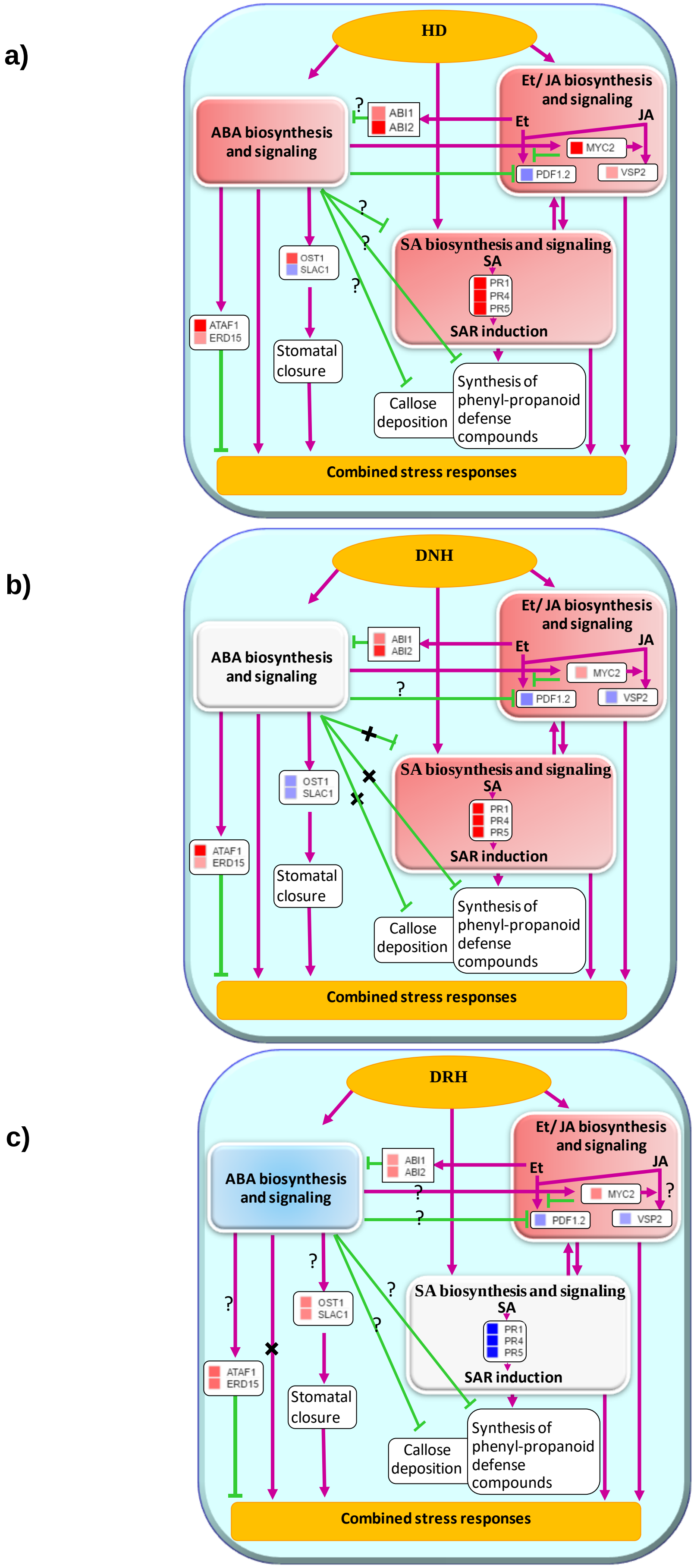

Supplementary Figure S13. Model representing the hormonal network under different combined stresses. The hormone and transcriptome profile data was integrated to redraw the model from Atkinson and Urwin (2012). The diagram depicts crosstalk between different hormones during combined stress. The role of plant hormones in regulating combined host pathogen and drought stress (HD) **(a)**, drought and non-host pathogen (DNH) **(b)** and host pathogen inoculated during drought recovery (DRH) **(c)** is presented based on the respective combined stress data. The figure is in continuation with **Figure 5**. Red colour boxes indicate elevation in hormone content and white boxes indicate no change and blue boxes indicate reduction over control. Purple arrows show induction or positive regulation, while green bars show inhibition or repression of gene or process. Cross signs represent inhibition of the step in absence of hormone or its signalling. Question mark presents the unknown trend in hormone content and signaling network. Pattern of gene expression is shown in the form of heatmap where red colour shows up- and blue colour shows down-regulation. ABA, abscisic acid; JA, jasmonic acid; SA, salicylic acid; PR, pathogenesis-related; SAR, systemic acquired resistance.

Supplementary table 1. Details of cross talk by three major hormones under drought and pathogen stress reported as individual stress studies in the literature*.

S. No.	Plant	Pathogen	Crosstalk	Phytohormone levels	Plant response	Reference
1.	*Arabidopsis thaliana*	*Pseudomonas syringae* (avirulent isolate)	SA-JA antagonism	SA signaling and suppression of JA signaling	Enhanced susceptibility to *Alternaria brassicicola*	[1]
2.	*A. thaliana*	*P. syringae* pv. tomato DC3000	ABA-SA antagonism	Increase in ABA levels and suppression in SA levels	Enhanced susceptibility	[2]
3.	*A. thaliana aao3* mutant	*P. syringae* pv. tomato DC3000	ABA-SA antagonism	Decreased ABA levels, increased SA levels	Reduced pathogen multiplication	[2]
4.	*Phillyrea angustifolia*	Drought	ABA-SA synergism	Increased SA levels	Drought stress tolerance	[3]
5.	*Hordeum vulgare*	Drought	ABA-SA synergism	Increased SA levels	Drought stress tolerance	[4]
6.	*A. thaliana aba2* mutant	*Pythium irregular*	ABA-JA synergism	Decreased JA levels	Suppression of SA signaling	[5]
7.	*A. thaliana*	*Alternaria brassicicola*	ABA-JA synergism	Increased ABA and JA levels	Resistance to the fungus	[6]
8.	*A. thaliana*	Drought	ABA-JA synergism	Increase in JA along with ABA	-	[7]
9.	*Brassica juncea*	Exogenous application of JA	-	-	Drought stress tolerance	[8]
10.	*Pyrus bretschneideri*	Exogenous application of JA	-	-	Drought stress tolerance	[9]
11.	*A. thaliana*	Exogenous treatment of ABA	ABA-JA antagonism	-	Susceptibility *Fusarium oxysporum*	[10]
12.	*A. thaliana*	SA at higher concentrations	-	-	Decreased drought tolerance	[11]
13.	*Cucumis melo*	SA pretreatment	-	-	Drought stress tolerance	[12]

14.	*Zea mays*	SA pretreatment	-	Enhanced activity of antioxidant enzymes, superoxide dismutase, ascorbate peroxidase, glutathione reductase and monodehydroascorbate reductase	Drought stress tolerance	[13]
15.	*A. thaliana*	Low concentrations of JA and SA	-	Increased transcript expression of JA responsive *PDF1.2* gene and SA-regulated *PR-1* gene	-	[14]

*Literature where the hormone quantification data available were considered. Studies reporting phytohormone crosstalk by experimental evidences were included. Table lists major synergistic and antagonistic crosstalk between ABA-SA-JA and suggests the existence of both scenarios (synergistic and antagonistic) between three hormones. Resultant influence of such crosstalk on plant response towards subsequent drought or pathogen stress is also listed here.

References

1. Spoel, S. H., Johnson, J. S. & Dong, X. Regulation of tradeoffs between plant defenses against pathogens with different lifestyles. *Proc. Natl. Acad. Sci. U.S.A.* **104**, 18842–18847 (2007).

2. De Torres-Zabala, M., Truman, W., Bennett, M. H., et al. *Pseudomonas syringae* pv. tomato hijacks the Arabidopsis abscisic acid signalling pathway to cause disease. *EMBO J*. **26**, 1434-1443 (2007).

3. Munne-Bosch, S. & Penuelas, J. Photo- and antioxidative protection, and a role for salicylic acid during drought and recovery in field-grown *Phillyrea angustifolia* plants. *Planta* **217**, 758–766 (2003).

4. Bandurska, H. & Stroiński, A. The effect of salicylic acid on barley response to water deficit. *Acta Physiol. Plant*. **27**, 379–386 (2005).

5. Adie, B. A. T, Pérez-Pérez, J., Pérez-Pérez, M. M., Godoy, M., Sánchez-Serrano, J. J., Schmelz, E. A. & Solano, R. ABA is an essential signal for plant resistance to pathogens affecting JA biosynthesis and the activation of defenses in Arabidopsis. *Plant Cell* **19**, 665–681 (2007).

6. Fan, J., Hill, L., Crooks, C., Doerner, P. & Lamb, C. Abscisic acid has a key role in modulating diverse plant-pathogen interactions. *Plant Physiol*. **150**, 1750-1761 (2009).

7. Harb, A., Krishnan, A., Ambavaram, M. M. R. & Pereira, A. Molecular and physiological analysis of drought stress in Arabidopsis reveals early responses leading to acclimation in plant growth. *Plant Physiol*. **154**, 1254–1271 (2010).

8. Alam, M., Nahar, K., Hasanuzzaman, M. & Fujita, M. Exogenous jasmonic acid modulates the physiology, antioxidant defense and glyoxalase systems in imparting drought stress tolerance in different Brassica species. *Plant Biotechnol*. *Rep*. **8**, 279–293 (2014).

9. Gao, X. P., Pan, Q. H., Li, M. J., Zhang, L. Y., Wang, X. F., Shen, Y. Y., Lu, Y. F., Chen, S. W., Liang, Z. & Zhang, D. P. Abscisic acid is involved in the water stress-induced betaine accumulation in pear leaves. *Plant Cell Physiol*. **45**, 742-750 (2004).

10. Anderson, J. P., Badruzsaufari, E., Schenk, P. M., Manners, J. M., Desmond, O. J. & Ehlert, C. Antagonistic interaction between abscisic acid and jasmonate-ethylene signaling pathways modulates defense gene expression and disease resistance in Arabidopsis. *Plant Cell* **16**, 3460–3479 (2004).

11. Borsani, O., Valpuesta, V. & Botella, M. A. Evidence for a role of salicylic acid in the oxidative damage generated by NaCl and osmotic stress in Arabidopsis seedlings. *Plant Physiol*. **126**, 1024–1030 (2001).

12. Korkmaz, A., Uzunlu, M. & Demirkiran, A. Treatment with acetyl salicylic acid protects muskmelon seedlings against drought stress. *Acta Physiol. Plant*. **29**, 503–508 (2007).

13. Saruhan, N., Saglam, & A. Kadioglu, A. Salicylic acid pretreatment induces drought tolerance and delays leaf rolling by inducing antioxidant systems in maize genotypes. *Acta Physiol. Plant*. **34**, 97-106 (2012).

14. Mur, L. A. J., Kenton, P., Atzorn, R., Miersch, O. & Wasternack, C. The outcomes of concentration-specific interactions between salicylate and jasmonate signaling include synergy, antagonism, and oxidative stress leading to cell death. *Plant Physiol*. **140**, 249–262 (2006).

Supplementary table 2. Summary of primer sequences used in RT-qPCR based validation of key hormone biosynthesis genes.

Gene ID	Oligo name	Oligo sequence (5' to 3')
AT1G74710	AtICS1-FP	CCTAATGGCAAGATCGCTGT
	AtICS1-RP	AGCAATAGTTGCAGCCAACA
AT3G10340	AtPAL4-FP	TTGCTTCCATTGGGAAACTC
	AtPAL4-RP	CGCCTTTAAACCCGTAATCA
AT2G06050	AtOPR3-FP	CGGCGTTGGCAGAGTATTAT
	AtOPR3-RP	TCCCTTAGCGTGAACTGCTT
AT5G42650	AtAOS-FP	ATGCCGTCAACGGAACTAAC
	AtAOS-RP	ACTCAGGGAAGATCCGGTTT
AT1G16540	AtABA3-FP	TAGTGGAGGCACTGTTGCTG
	AtABA3-RP	GCATCCAAATTGCAGAAGGT
AT3G14440	AtNCED3-FP	GGCTTGGTGGCAATCATACT
	AtNCED3-RP	GAGTGTGAAGCGCAGATGAA
AT2G37040	AtPAL1-FP	AGCCACACATTGCCACACTC
	AtPAL1-RP	ATCTCCGGAGGCGGTGATTG
AT3G53260	AtPAL2-FP	TCACCGGCCGTCCTAATTCC
	AtPAL2-RP	CGCCGTGCCATTAACGAGAG
